# Heat and Pressure Treatments on Almond Protein Stability and Change in Immunoreactivity after Simulated Human Digestion

**DOI:** 10.3390/nu10111679

**Published:** 2018-11-05

**Authors:** Elisabetta De Angelis, Simona L. Bavaro, Graziana Forte, Rosa Pilolli, Linda Monaci

**Affiliations:** Institute of Sciences of Food Production, National Research Council of Italy (ISPA-CNR), Via Amendola 122/O, 70126 Bari, Italy; elisabetta.deangelis@ispa.cnr.it (E.D.A.); simona.bavaro@ispa.cnr.it (S.L.B.); grazianaforte@hotmail.it (G.F.); rosa.pilolli@ispa.cnr.it (R.P.)

**Keywords:** almond, thermal/pressure treatment, autoclave, food allergens, High Resolution Mass Spectrometry (HR-MS), immunoreactivity reduction, in vitro digestion

## Abstract

Almond is consumed worldwide and renowned as a valuable healthy food. Despite this, it is also a potent source of allergenic proteins that can trigger several mild to life-threatening immunoreactions. Food processing proved to alter biochemical characteristics of proteins, thus affecting the respective allergenicity. In this paper, we investigated the effect of autoclaving, preceded or not by a hydration step, on the biochemical and immunological properties of almond proteins. Any variation in the stability and immunoreactivity of almond proteins extracted from the treated materials were evaluated by total protein quantification, Enzyme Linked Immunosorbent Assay (ELISA), and protein profiling by electrophoresis-based separation (SDS-PAGE). The sole autoclaving applied was found to weakly affect almond protein stability, despite what was observed when hydration preceded autoclaving, which resulted in a loss of approximately 70% of total protein content compared to untreated samples, and a remarkable reduction of the final immunoreactivity. The final SDS-PAGE protein pattern recorded for hydrated and autoclaved almonds disclosed significant changes. In addition, the same samples were further submitted to human-simulated gastro-intestinal (GI) digestion to evaluate potential changes induced by these processing methods on allergen digestibility. Digestion products were identified by High Pressure Liquid Chromatography-High Resolution Tandem Mass Spectrometry (HPLC-HRMS/MS) analysis followed by software-based data mining, and complementary information was provided by analyzing the proteolytic fragments lower than 6 kDa in size. The autoclave-based treatment was found not to alter the allergen digestibility, whereas an increased susceptibility to proteolytic action of digestive enzymes was observed in almonds subjected to autoclaving of prehydrated almond kernels. Finally, the residual immunoreactivity of the GI-resistant peptides was in-silico investigated by bioinformatic tools. Results obtained confirm that by adopting both approaches, no epitopes associated with known allergens survived, thus demonstrating the potential effectiveness of these treatments to reduce almond allergenicity.

## 1. Introduction

Tree nuts are cultivated and consumed around the world due to their pleasant taste and nutritional/health properties, and among these almond (*Prunus dulcis* (Mill.) D. A. Webb or *Amygdalus communes* L.) represents one of the most commonly consumed [1]. Almond is considered a valuable source of lipids (mainly represented by monounsaturated fatty acids), proteins, dietary fibers, vitamins (e.g., vitamin E), minerals, phenolic compounds, and phytosterols [2,3,4]. Globally, in 2016 America represented the main almond producer (63%) followed by Asia (16%), Europe (10%), Africa (9%) and Oceania (2%) [5]. Despite its economic and health importance, almond is renowned for triggering immunological reactions in sensitive individuals. Indeed, according to studies on the prevalence of tree nuts allergies, almond allergy usually ranks fourth [6,7]. So far, eight groups of allergens have been identified in almonds, namely Pru du 1, Pru du 2, Pru du 2S albumin, Pru du 3, Pru du 4, Pru du 5, Pru du 6, and Pru du γ-conglutin. Among these eight groups, only Pru du 3 (nsLTP), Pru du 4 (profilin), Pru du 5 (60 S ribosomal protein) and Pru du 6 (legumin) are recognized and included in the WHO−IUIS list of allergens [8]. Pru du 6, also named amandin or prunin, accounts for about 70% of the total soluble proteins and being the major almond protein component as well as its major almond allergen [9,10]. Pru du 6 is a hexameric protein comprising six subunits with a total molecular weight of about 360 kDa. By isolating and sequencing cDNA clones from almond, it has been inferred that prunin consists of two seed storage proteins of 61.0 and 55.9 kDa, named prunin-1 (Pru-1) and prunin-2 (Pru-2), respectively, that are assembled by means of disulfide bonds [11,12]. Both Pru-1 and Pru-2 have two polypeptides linked by disulfide bonds. Specifically, Pru-1 is composed of an acidic α-chain of 40.1 kDa (pI = 5.4) and a basic β-chain of 20.9 kDa (pI of 9.6). While Pru-2 is divided into two subunits of 34.5 kDa (pI = 4.6) and 21.4 kDa (pI = 9.5), corresponding to the α- and β-chains, respectively [11]. Pru-1 is highly water-soluble and it has been recently identified as the major component of almond prunin [12]. Several studies demonstrated that prunin was thermally stable, suffering from partial unfolding only at temperatures >94 °C. In addition, it tends to aggregate to food matrix producing different structures. In the presence of water, prunin easily denaturates with consequential decrease of its allergenicity [1]. 

Generally, almond can be consumed either raw (snacks) or processed and as ingredient of several food products (spreads, bakery, pastry, chocolates, and confectionary products) [13]. As ingredient and food allergen, almond could be inadvertently present in food as a result of cross contamination or production error, representing a risk for sensitized and/or allergic individuals. For this reason, a strict labeling regulation has been put in place in Europe [14] which imposes the obligatory label for 14 allergenic ingredients, among of which are tree nuts. So far, strict avoidance of allergenic proteins remains the most effective mean to prevent the occurrence of allergic reactions. In this scenario, several analytical methods, relied on the most advanced techniques, have been developed to keep under control food manufacturing chain and prevent accidental episodes of allergenicity [15,16,17,18]. In addition, the development of new strategies for allergenicity reduction, could represent a good alternative to protect allergic consumers’ health. A variety of foods (almonds included) are submitted to different processes before their consumption that may entail some changes in food proteins, including unfolding, aggregation or chemical modification which can significantly affect the final proteins immunoreactivity [19]. Different strategies were investigated to reduce almond allergenicity, including microwave heating [20,21], thermal processing [20,21,22,23], chemical processing [24], gamma irradiation [25] with partial alteration or no reduction in almond allergenicity. Recently, pulsed ultraviolet light and high pressure were demonstrated to significantly reduce prunin immunoreactivity [22,26,27]. Typically employed in sterilization procedure, autoclaving treatments (mainly performed at 121 °C, −1 atm) were largely investigated for its potential to alter the intrinsic almond allergenicity. However, scarce results were obtained [9,20,21,25] with the only exception shown for almonds autoclaved in presence of water [22]. 

Resistance to digestion by gastro-intestinal (GI) protease represents another important parameter to consider when assessing the residual immunoreactivity of a protein. To sensitize an individual via the GI tract, an allergen must preserve its structure during digestion process, thus allowing the intact epitopes to be taken up by the gut to sensitize the mucosal immune system. Therefore, an assessment of the stability of a protein along digestion is important to understand its potential to trigger an immunoreaction [28]. In vitro digestion models simulating the human digestion process represent a useful tool to address this issue. The main advantages characterizing such approach are simplicity, low costs (compared to in vivo tests) and good reproducibility [29]. Moreover, the use of in vitro digestion models to test protein allergenicity was also recommended by the European Food Safety Authority (EFSA) panel [30]. As far as almond is concerned, several papers are present in literature tracking the fate of several almond components upon simulated human digestion: most of them focus on the study of almond lipid bioaccessibility, where natural, processed almonds (roasted and blanched) or almond by products (butter or muffin) were analyzed [31,32,33,34,35]. Polyphenols release from almond skin incorporated or not in food matrix along simulated human digestion was also deeply investigated [36,37]. Conversely, only a few papers explored the digestibility of almond proteins during simulated GI digestion, with the most recent represented by Toomer et al., 2013 and Mandalari et al., 2014 [38,39].

With the final aim to develop an effective technological strategy to reduce almond allergenicity, in the present work we investigated the effect of autoclave treatment, preceded or not by a hydration step, and performed at harsh conditions (134 °C and 2 atm), on the final immunoreactivity of almond seeds. The stability of almond proteins was evaluated by electrophoretic separation and any change in their final immunoreactivity was assessed by Enzyme Linked Immunosorbent Assay (ELISA) assay. In addition, autoclaved almonds were submitted to a standardized static in vitro digestion protocol and any alteration in the final allergen proteins digestibility, was investigated by SDS-PAGE and HPLC-MS/MS analysis. Ultimately, with the aid of online bioinformatics tools, the low molecular weight fraction of the GI digest was screened, looking for resistant peptides encrypting full-length linear epitopes that survived enzymatic proteolysis, thus assessing in-silico the potential residual immunogenicity of autoclaved almonds.

## 2. Materials and Methods 

### 2.1. Chemicals

Peeled raw almonds kernels (*Prunus dulcis* (Mill.) D. A. Webb, syn. *Prunus amygdalus* A. J. G. K. Batsch, var. California, CA, USA) were obtained from Besana S.p.A. (San Gennaro Vesuviano (NA), Italy). Sodium chloride, Trizma-base, urea, ammonium bicarbonate (AMBIC), iodoacetamide (IAA), dithiothreitol (DTT), along with chemicals for electrophoresis, namely sodium dodecyl sulfate-SDS, glycine, glycerol, Coomassie Brilliant Blue-G 250 were provided by Sigma-Aldrich (Milan, Italy). Methanol (HPLC grade) was obtained from VWR International S.r.l. (Milan, Italy) while acetonitrile (Gold HPLC ultragradient), trifluoroacetic acid (TFA) and Bromophenol blue were purchased from Carlo Erba Reagents (Cornaredo, Milan, Italia). Ultrapure water used was produced by a Millipore Milli-Q system (Millipore, Bedford, MA, USA). Formic acid (MS grade) was purchased from Fluka (Milan, Italy) while filters in Polytetrafluoroethylene (PTFE) from 0.45 µm were purchased from Sartorius (Göttingen, Germany) and syringe filters in cellulose acetate (CA) 1.2 µm from Labochem Science S.r.l. (Catania, Italy). Trypsin (proteomic grade) for in-gel protein digestion was purchased from Promega (Milan, Italy). As for in vitro digestion experiments, potassium chloride (KCl), potassium dihydrogen phosphate (KH_2_PO_4_), sodium bicarbonate (NaHCO_3_), sodium chloride (NaCl), magnesium chloride hexahydrate ((MgCl_2_(H_2_O)_6_), ammonium carbonate ((NH_4_)_2_CO_3_), sodium hydroxide (NaOH), hydrochloric acid (HCl) and calcium chloride (CaCl_2_) along with other chemicals and enzymes (salivary α-amylase, pepsin, trypsin, chymotrypsin, pancreatic α-amylase, pancreatic lipase plus phospholipid, bile, serine protease inhibitor (PMSF = methyl-phenyl-sulfonyl fluoride) were obtained from Sigma-Aldrich (Milan, Italy). 

### 2.2. Autoclave Processing

A total of 8 raw almond seeds (corresponding to a total weight of approximately 10 ± 0.5 g) were placed in a centrifuge tube and then submitted to autoclaving treatment. Two different processing schemes were investigated (i) autoclaving and (ii) sample prehydration followed by autoclaving. The hydration step was performed by adding 50 mL of ultrapure water to raw almond kernels followed by 2 h of shaking at room temperature in an orbital shaker (KS 4000 i-control shaker, IKA Works GmbH & Co. KG, Staufen, Germany). Water was discarded before autoclaving. Autoclave treatments were set as follows: temperature at 134 °C at the pressure of 2 atm and two time intervals were explored, namely 10 and 20 min. The system took about 40 min to reach the final temperature of 134 °C. In summary, four different treatments were studied:

(a) Almond autoclaved for 10 min (AC10),
(b) Almond autoclaved for 20 min (AC20),
(c) Almond prehydrated + autoclaved for 10 min (H_2_O_AC10),
(d) Almond prehydrated + autoclaved for 20 min (H_2_O_AC20).

As positive control, raw almonds not undergoing any treatment was also included in the study (CTRL).

### 2.3. Protein Extraction and Quantification

After treatment, raw and processed almond kernels were milled by using an electric miller (Mulinex, Milan, Italy) and 1.2 g of flour were extracted by adding 30 mL of TBS (50 mM Tris-HCl, 150 mM NaCl, pH 8) buffer containing 1 M Urea. Samples were left shaking for 2 h at room temperature in an orbital shaker (KS 4000 i-control shaker, IKA Works GmbH & Co. KG, Staufen, Germany) and then centrifuged for 15 min at 3082× *g* at 4 °C. The upper phase was discarded, and the supernatant was carefully collected and filtered through 1.2 µm CA syringe filters. The total protein content of raw and autoclaved almonds was calculated by Bradford assay (Quick Start™ Bradford Protein Assay, Bio-Rad Laboratories s.r.l., Segrate MI, Italy) that was accomplished according to the manufacturer’s instruction. Bovine serum albumin (BSA, 0.125–1 mg/mL) was used as the reference standard. Samples were stored at −20 °C until its use and filtered through 0.45 µm PTFE filters just before electrophoretic analysis. A post-hoc Tukey-Kramer test was performed to evaluate any statistically significant differences between protein levels estimated in untreated and treated almonds.

### 2.4. Sandwich ELISA for Almond Immunoreactivity

Immunoreactivity of almond allergens in processed and unprocessed samples was determined by using a commercially available almond ELISA kit (RidaScreen Fast/Almond, R-Biopharm AG, Darmstadt, Germany). Kit instructions were followed and three replicates of the controls and the samples previously diluted 1:10,000 were plated. Absorbance values (*λ* = 450 nm) were read on a microplates reader (BioTek Instruments Inc., Winooski, VT, USA) and finally Tukey-Kramer test was performed to evaluate any statistically significant difference in the results obtained. 

### 2.5. Almond In Vitro Digestion 

Raw and selected treated almond flours, were successively subjected to in vitro simulated human digestion according to a standardized static model proposed by Minekus et al. in 2014 with chew, gastric and duodenal digestion mimicking the physiological conditions [29]. Simulated salivary fluid (SSF, pH 7), simulated gastric fluid (SGF, pH 3), and simulated intestinal fluid (SIF, pH 7) were prepared according to the harmonized conditions. The whole digestion procedure was accomplished according to the protocol described by Bavaro et al., 2018 [40]. As for duodenal phase, bile salts were added and single enzymes (trypsin, chymotrypsin, pancreatic lipase, and pancreatic α-amylase) were used in alternative to pancreatin. The reaction was stopped by addition of a protease inhibitor (phenylmethylsulfonyl fluoride) and the resulting digests were centrifuged at 2360× *g* for 5 min at 4 °C. The collected supernatant was stored at −20 °C until further analysis. A parallel experiment was carried out by subjecting untreated almonds to GI fluids (SSF, SGF and SIF) without the addition of enzymes, to assess the proteins extractable by digestive fluids that would represent the amount to be digested. In summary the samples obtained after GI digestion, with or without the addiction of enzymes were the following:

(a) untreated almonds submitted only to biological fluids, no enzymes (CTRL-NE),
(b) untreated almonds undergoing the whole GI digestion (CTRL-GI),
(c) AC10 almonds subjected to complete GI digestion (AC10-GI),
(d) H_2_O-AC10 almonds subjected to the whole GI digestion (H_2_O-AC10-GI).

### 2.6. Electrophoretic Analysis of Almond Proteins

Ten micrograms of protein were extracted from raw and treated almonds, along with supernatants aliquots of the gastric and duodenal digesta (obtained by adding or not the enzymes in SSF, SGF, SIF) and separated under reducing conditions, by means of sodium dodecyl sulfate-polyacrylamide gel electrophoresis (SDS-PAGE) on 8–16% polyacrylamide pre-cast gels (13.3 cm × 8.7 cm × 1.0 mm) using a Criterion™ Cell equipment (Bio-Rad Laboratories, Segrate, MI, Italy). While whole protein extracts were submitted to electrophoresis separation without any preliminary treatment, digestive fluids were purified by means of ReadyPrep™ 2-D Cleanup Kit (Bio-Rad Laboratories, Segrate, MI, Italy) before separation, to remove lipid and saline components which could interfere with SDS-PAGE analysis. Digests clean-up was performed according to the manufacturer’s instruction. Before electrophoresis analysis, samples were denatured with Laemmli buffer (62.5 mM Tris-HCl, pH 6.8, 25% glycerol, 2% SDS, 0.01% Bromophenol Blue, 100 mM DTT) (1:1 ratio) for 5 min at 100 °C. As running buffer, a TGS (25 mM Tris, 192 mM Glycine, 0.1% SDS) solution was used. Electrophoretic separation was performed at 60 V for the first 20 min and then at 100 V until the end of the run. Finally, gels were stained with Coomassie Brilliant Blue-G-250 solution and the protein profiles detected on a ChemiDoc™ Imaging System (Bio-Rad Laboratories, Segrate, MI, Italy). Precision Plus Protein^TM^ all blue standards (10–250 kDa, Bio-Rad Laboratories, Segrate, MI, Italy) was used as protein reference for molecular weight.

### 2.7. In-Gel Tryptic Digestion

The most relevant protein bands detected along the electrophoretic gel of almond samples submitted to GI in vitro experiments, including or not digestive enzymes, were excised from the polyacrylamide gels and in-gel trypsin digested according to the protocol described by De Angelis et al., 2017 [41]. After drying, each sample was resuspended in 100 µL of H_2_O/ACN 95/5 + 0.1% formic acid (*v/v*) and 20 µL were injected into LC/MS apparatus.

### 2.8. Separation of Low Molecular Weight Fractions of Duodenal Samples

Polypeptides and small peptides produced by submitting untreated and processed almonds to simulated GI digestion, were separated via size exclusion chromatography by passing samples through Bio-Spin^®^6 Tris Columns (Bio-Rad Laboratories, Segrate, MI, Italy) whose cut-off is around 6 kDa. Specifically, after column conditioning (addition of 500 µL of H_2_O + 0.1% FA to the column and centrifugation at 1000× *g* for 1 min, repeated for 5 times) 100 µL of sample were loaded onto the column and centrifuged for 4 min at 1000× *g* to collect the protein fraction with a molecular weight higher than 6 kDa. Low molecular weight components (<6 kDa) were withdrawn by washing the column with 100 µL of H_2_O + 0.1% FA (addition of solvent to the column and centrifugation at 1000× *g* for 4 min). This procedure was repeated twice, and the eluted volumes were pooled together (total volume 200 µL) and dried up to the final volume of 100 µL. Finally, extracts were filtered through a cellulose syringe filter (0.45 μm) and stored at −20 °C before untargeted LC-HRMS/MS analysis. Samples were diluted 1:1 (*v/v*) with H_2_O + 0.1% FA just before LC/MS analysis.

### 2.9. Untargeted HPLC-HRMS/MS Analysis

HPLC-MS/MS data were acquired on a Q-Exactive™ Plus Hybrid Quadrupole-Orbitrap™ Mass Spectrometer coupled with a Ultra High Performance Liquid Chromatography (UHPLC) pump system (Thermo Fisher Scientific, Bremen, Germany). Peptides mixture obtained from protein bands in-gel digested referred to samples CTRL-NE, CTRL-GI, AC10-GI and H_2_O-AC10-GI, along with the low molecular weight molecules arisen from raw and treated almonds completely digested (CTRL-GI, AC10-GI and H_2_O-AC10-GI), were separated on a reversed phase Aeris peptide analytical column (internal diameter 2.1 mm, length 150 mm, particle size 3.6 µm, porosity 100 Å, Phenomenex, Torrance, CA, USA) at a flow rate of 200 µL/mL. The elution gradient used for peptide separation was the following: from 0–50 min solvent B increased from 5% to 60%, 50–51 min further increase from 60% to 80%, then kept constant for 13 min, 63–80 min at a constant 5% for column conditioning before next injection. Solvent A = H_2_O + 0.1% FA, solvent B = Acetonitrile + 0.1% FA. Volume injection was set to 20 µL and each sample was injected twice in MS. Spectra were acquired in the mass range of 180–2000 *m/z* by applying the data dependent (FullMS-dd2) acquisition mode analysis and only positive ions were considered. Other MS parameters were the same as described in Bavaro et al., 2018 [40], with exception of dd-setting maximum Automatic Gain Control (AGC) target value that was set here at 5.00 e1. Moreover, in the current work, ions with charge higher than 4 were excluded. 

MS data were then simultaneously processed via the commercial software Proteome Discoverer^TM^ version 2.1.1.21 (Thermo-Fisher-Scientific, San José, CA, USA) and protein identification was achieved by SequestHT search against a customized database including almond proteins extracted by Swiss Prot DB on the base of the taxonomy code of *Amygdalus dulcis* (ID: 3755, containing about 450 sequences), along with the sequences of all specific enzymes used for GI digestion. Due to the complexity of enzyme mixtures used for gastro-duodenal digestion simulation, an unspecific cleavage was set for peptide identification of low molecular weight fraction of GI samples. For other samples, trypsin was selected as cleavage enzyme. In all cases, mass tolerance on the precursor and fragment ions was set to 5 ppm and 0.05 Da, respectively. Moreover, only trustful peptide-spectrum matches were accepted and in particular a minimum of three peptides was set as threshold for protein identification, after filtering the peptide list to the sequences assigned with at least medium confidence (False Discovery Rate—FDR < 5%).

### 2.10. Bioinformatics Analysis for Assessing the Residual Immunoreactivity of Almond Proteins after GI Digestion

Peptide sequences identified in the low molecular weight fraction of duodenal digests referred to untreated and processed samples were finally screened in Immune Epitope Database (IEDB database) online platform, to detect epitope linear sequences surviving gastro-duodenal digestion. The IEDB results were filtered as follows: linear sequence for epitope structure, exact match for Basic Local Alignment Search Tool (BLAST) option and human as host.

## 3. Results and Discussion

In the current study a common food processing treatment based on the combined effect of heat and pressure, namely autoclaving, was investigated on almond seeds, with the final aim to reduce their allergenic potential. Almond kernels were submitted to two autoclaving schemes differing in the presence, or absence, of a preliminary hydration step (for 2 h) before autoclaving. Samples were autoclaved at the temperature of 134 °C, pressure of 2 atm for 10 or 20 min in both schemes. Any changing in protein solubility, because of thermal/pressure treatments, was assessed by estimating the almond protein contents with a Bradford assay, as previously reported in other works [27,40]. In addition, the direct comparison of the SDS-PAGE profiles of treated and untreated almonds provided information about the proteins mainly involved in the autoclave-induced modification. Then, a commercial sandwich ELISA kit against almond proteins was used to evaluate the variation in the total immunoreactivity of almonds after the thermal/pressure treatments explored. Finally, we tracked the fate of almond proteins differently processed upon in vitro simulated human gastro-duodenal digestion by a static digestion model. The residual immunoreactivity of peptides arisen from GI digests was finally estimated in-silico by bioinformatic tools. 

### 3.1. Effect of Thermal/Pressure Treatments on Solubility/Content of Almond Proteins 

**Figure 1:**
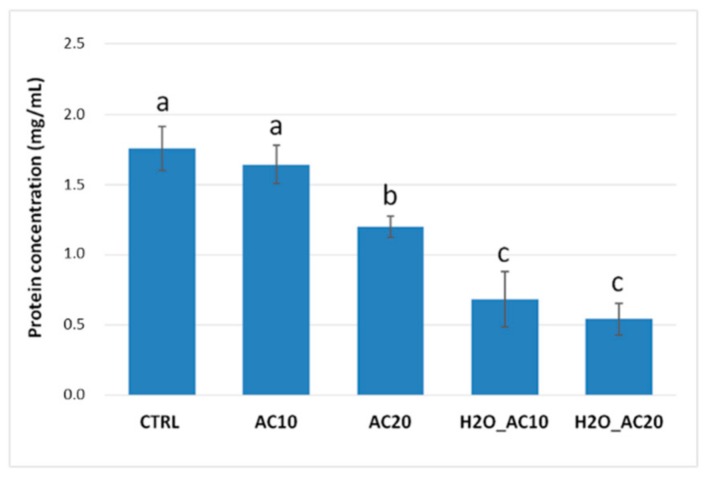
Protein content estimated with Bradford protein assay corresponding to untreated almond (CTRL) and almond submitted to autoclaving for 10 min (AC10) and 20 min (AC20), prehydration/autoclaving for 10 min (H_2_O_AC10) and 20 min (H_2_O_AC20). The results of a Tukey-Kramer test for multiple mean comparison are also reported and expressed using the following annotation a = CTRL and almonds autoclaved for 10 min (no statistically significant difference found); b = almonds autoclaved for 20 min; c = almonds hydrated and autoclaved for 10 and 20 min (no statistically significant difference found).

As well known, food processing can often cause some changes in proteins structures with a resulting decrease in their solubility, and the extent of these phenomena largely depends on the severity and duration of the process. Besides, autoclaving treatments may affect protein stability, modifying their final solubility. To have more insights on this, the protein content of raw and autoclaved almonds submitted to the different schemes, was estimated by Bradford assay, and compared each other. Results are displayed in Figure 1. For 10 min (AC10) provided a relative protein recovery similar to the untreated samples’ one. Whereas, the recovery was significantly reduced when autoclaving was prolonged up to 20 min (AC20), in this case a reduction by 30% was calculated compared to the untreated sample. A higher loss in protein recovery was observed in almonds kernels submitted to hydration before autoclaving. In fact, protein content dropped down to 30% after combination of prehydration/autoclaving for 10 min (H_2_O_AC10) compared with the raw almonds, and this trend remained constant also extending the treatment up to 20 min (H_2_O_AC20). Our results are in accordance with those described by Zhang et al., 2016 [22] who investigated the changes in the solubility and immunological properties of almond proteins submitted to different heat and pressure treatments, including dry/moist heat, autoclave sterilization (121 °C, 0.15 MPa) and high-pressure treatment, each tested under different conditions. For autoclaving experiments, they found a little change in protein solubility after 10 min of treatment and a clear decrease in protein recovery in samples autoclaved in presence of Phosphate-buffered saline (PBS), suggesting that the presence of water, in combination with heat and pressure applied, enhanced such reduction in protein solubility. A decrease in almond protein solubility due to boiling and autoclaving was already reported by Venkatachalam (2002) [21]. These phenomena were explained by considering the numerous biochemical and structural modifications that proteins underwent during heat and pressure treatments. It should be hypothesized that this processing cause protein unfolding due to the loss of secondary and tertiary structures. In addition, precipitation or aggregation phenomena due to the formation of intra- or inter-molecular covalent and non-covalent interactions between proteins or protein-food matrix could occur, with a consequent decrease in protein solubility [19]. The general decrease in protein content observed in treated almonds (Figure 1) demonstrated that autoclave-based treatment altered somehow the structure of almond proteins promoting a reduction of their solubility, and this effect appears even more enhanced by preceding autoclaving with exposition to water.

### 3.2. Impact of Thermal/Pressure Process on Immunoreactivity of Almond Proteins by ELISA Assay

**Figure 2:**
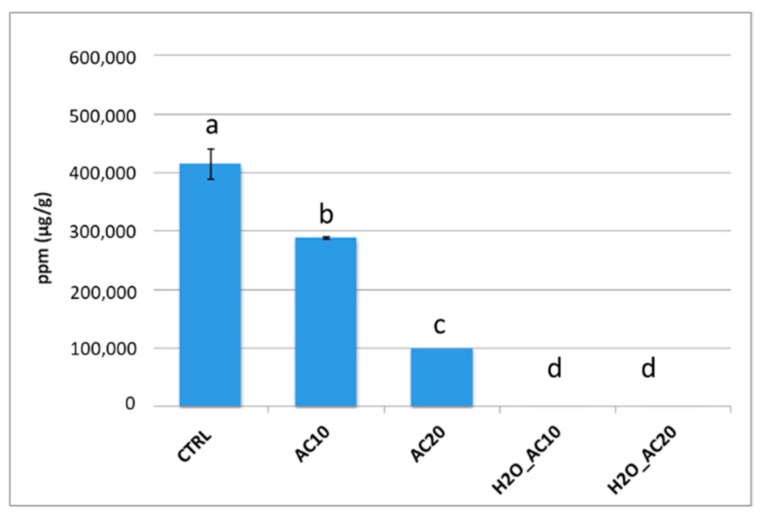
Immunoreactivity of almond proteins estimated by ELISA referred to raw (CTRL), autoclaved samples for 10 (AC10) and 20 min (AC20) and prehydrated and autoclaved samples for 10 (H_2_O_AC10) and 20 min (H_2_O_AC20) at 134 °C, 2 atm. The results of a Tukey-Kramer test for multiple mean comparison are also reported and expressed using the following annotation a = CTR; b = almonds autoclaved for 10 min; c = almonds autoclaved for 20 min; d = almonds hydrated and autoclaved for 10 and 20 min (no statistical difference found).

Food processing is also renowned to affect protein allergenic potential. Indeed, the numerous chemical and structural modifications that proteins underwent during processing techniques, could result in a destruction, masking or unmasking of conformational epitopes, thus altering the final food immunoreactivity [19]. In light of this, the effect of autoclaving process (accomplished with or without incubation with water) on the final allergenicity of almonds was firstly assessed via commercial sandwich ELISA kit (RidaScreen Fast/Almond, R-Biopharm, Darmstadt, Germany). Due to the lack of manufacturer’s information about the almond allergen which the antibody is raised against, the levels of immunoreactivity recorded for each sample were considered representative of the total allergenicity of the food tested. The histograms in Figure 2 illustrated the ELISA results obtained.

With respect to untreated almonds (CTRL) where a very high reactivity was recorded, a general decrease in the IgG reactivity was observed after autoclaving. In particular, an immunoreactivity reduction by 30% and 75% was displayed for the almonds AC10 and AC20, respectively. On the contrary, a minimal response antigen-antibody was recorded for prehydrated/autoclaved samples at both times investigated. 

Several papers [9,21,22] reported the effect of autoclave processing applied to almond, with negligible effects on the final allergenicity. Venkatachalam et al., in 2002, reported that submitting almonds to autoclaving at 121 °C, 1 atm for different time lengths (5–30 min) was not sufficient to produce a consistent reduction in almond allergenicity [21]. In a previous work, Roux et al. in 2001 had observed that although prunin content was reduced within first minutes of autoclaving (121 °C/1 atm, 2–60 min), the total allergenicity of almond protein extract remains constant. Interestingly, they found that after prolonging autoclaving for 40 and 60 min a more intense signal was highlighted in the higher molecular weight area in the Western blot, suggesting heat-induced protein aggregation phenomena [9]. Zhang et al. (2016) further confirmed that autoclaving treatment (121 °C, 0.15 MPa, 10 min) was not able alone to produce a significant reduction in almond allergenicity [22]. Conversely to previously reported, we observed a consistent reduction in IgG response in our autoclaved samples by approximately 75% if the treatment was kept for 20 min (Figure 2). This result might be due to the harsher autoclaving conditions (134 °C, 2 atm) applied in our experiments. Concerning prehydrated autoclaved almonds (Figure 2, H_2_O_AC10, H_2_O_AC20), we observed that immunoreactivity levels dropped down to a minimum detectable level, suggesting that soaking kernels with water before treatment could promote a better displacement of allergenic proteins or, alternatively, promote their aggregation masking the epitopes that are then no longer available for IgG binding. In this regard, Zhang et al. (2016) found similar results in defatted flour almond autoclaved in presence of PBS, inferring that higher temperature and pressure applied during autoclaving in presence of water produced a greater loss of immunoreactivity [22]. Autoclaving preceded by water incubation was successfully investigated also for allergenicity reduction in peanuts [40].

### 3.3. Protein Profiling of Untreated and Autoclaved Almond by SDS-PAGE Analysis

**Figure 3:**
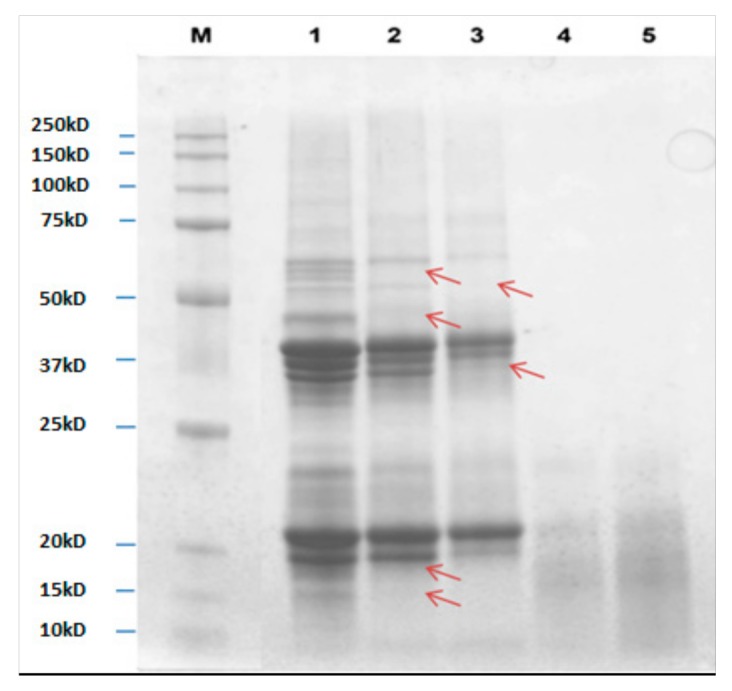
Comparison between SDS-PAGE protein profiles of almonds untreated (lane 1), autoclaved for 10 (lane 2) and 20 min (lane 3) and prehydrated and autoclaved for 10 (lane 4) and 20 min (lane 5) at 134 °C, 2 atm. M: MW reference standard.

The protein/peptides profiles of untreated and autoclaved (with or without a prehydration step) almonds at different times were compared in Figure 3, and the respective differences were marked with arrows. For each sample, a quantity of proteins equal to 10 µg was loaded onto the gel. As known from the literature, in absence of reducing agent, the major almond allergen, prunin (Pru du 6) has two major polypeptides with estimated molecular weights (MW) of 61 and 63 kDa, namely prunin 1 (Pru-1) and prunin 2 (Pru-2). Each polypeptide is composed of an acidic subunit (42–46 kDa) and a basic subunit (20–22 kDa) linked by disulfide bonds [10]. 

In the presence of DTT reagent, acid and basic subunits of Pru-1 and Pru-2 are released and they can be clearly seen in the lanes of untreated samples (CTRL, lane 1). Other bands are visible over 50 kDa region and below 20 kDa, these latter are likely to be attributed to Pru du 4 and Pru du 5 which MWs were reported to be approximately 14 and 11 kDa, respectively. In 10 min autoclaved almonds (AC10, lane 2), a general decrease in bands intensity was displayed, with a concomitant disappearance of some signals in the 60 kDa region and below 50 kDa and 20 kDa. By prolonging autoclaving to 20 min (AC20, lane 3), a further reduction in some band’s signals (25–50 kDa and 20–25 kDa regions) as well as a clear disappearance of two bands with MW approximately of 52 and 37 kDa were observed. On the contrary, protein profiles referring to samples autoclaved for 10 and 20 min after incubation with water (H_2_O-AC10, lane 4, H_2_O-AC20, lane 5) appeared as a smear of peptides with low MW (15–20 kDa), probably produced by fragmentation phenomena occurring during the applied treatments.

Results obtained by SDS-PAGE analysis agree with those obtained by ELISA assay, where a gradual reduction of allergenicity of almonds autoclaved for 10 and 20 min (30 and 75%, respectively), followed by a drastic drop of IgG response in prehydrated autoclaved samples, was pointed out. As previously discussed, protein bands comprised between 25 and 50 kDa, along with those ranging around at 20–22 kDa, were putatively attributed to acidic and basic subunits of Pru-1 and Pru-2 polypeptides that composed Pru du 6. This is the most abundant protein in almond and represents the main allergen of this nut. In the light of this, it is reasonable to assume that, in samples AC10 and 20 min, the gradual signal decrease of these bands was caused by a gradual reduction of Pru du 6 content, which could explain the decrease of immunoreactivity recorded during ELISA test in the same samples. Although with a reduced content, Pru du 6 bands persisted after 20 min of autoclaving, confirming the thermostable nature of this protein [42]. The allergenicity decrease observed in autoclaved almonds, could be due to the degradation of Pru du 4 (14 kDa) and Pru du 5 (11.4 kDa) induced by this processing, as demonstrated by the disappearance of the corresponding bands along the AC10 and AC20 profiles. 

As for autoclaved almonds pre-incubated with water, only small peptides were observed along the SDS-PAGE profile, suggesting that Pru du 6 was completely degraded by the treatment applied with a consequent decrease in the final allergenicity, proved by the low reactivity detected in ELISA test. 

Protein degradation and fragmentation induced by autoclaving was already reported in literature by Cabanillas et al. (2014, 2015) and Bavaro et al. (2018) on walnuts and peanuts, respectively [40,43,44]. Similar to that reported in the present work, Bavaro et al. explored the effect of the prehydration before autoclaving on peanut and they obtained a similar reduction in IgG immunoreactivity of processed peanuts. They explain these phenomena by considering that water absorbed by seeds facilitated heat propagation in the inner part of the seed, as well as exert a mechanical effect during high-pressure autoclaving, which promoted the disaggregation and decrease in spot intensity. It is not excluded that the allergenicity reduction observed in autoclaved food should be attributable to a loss of proteins solubility, likely induced by the several structural changes (conformational changes in the protein, formation of intra and/or inter-molecular covalent and non-covalent interactions, etc.) promoted by the combination of heating and pressure. However recent studies have demonstrated that extensive proteins solubilization of the pressure/heated food materials produces the same SDS-PAGE profile of protein degradation, with an overall decreased of the response antigen-antibody [40,43].

### 3.4. Fate of Heat/Pressured Almonds Proteins upon In Vitro Gastro-Duodenal Digestion and Evaluation of Residual Immunoreactivity 

The effect on the biochemical and structural modification occurring on proteins undergoing food processing may largely affect their susceptibility to gastro-duodenal digestion, absorption kinetics and consequently the allergic response of the immune system. In this section, we investigated whether autoclaving (including or not the preliminary water incubation) might alter almond proteins digestibility by performing in vitro simulated human gastro-duodenal digestion experiments. Finally, the residual immunoreactivity of the final digests was evaluated by bioinformatic approach.

#### 3.4.1. Digestibility of Almond Proteins upon Simulated Gastric and Duodenal Digestion 

**Figure 4:**
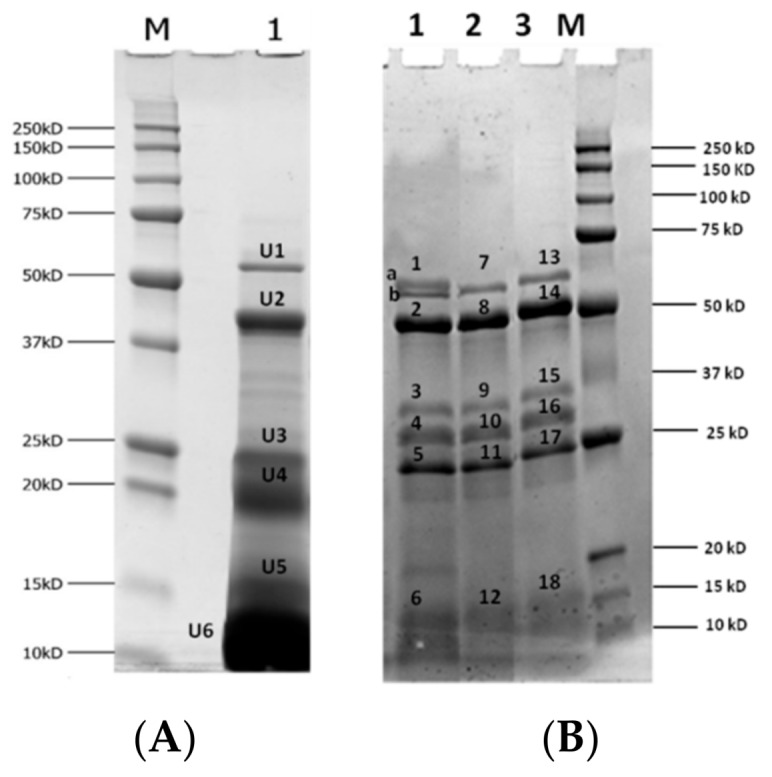
(Panel **A**) SDS-PAGE protein profile of raw almonds submitted to chew, gastric and intestinal environments without adding enzyme mixture. Lane 1: untreated almond. (Panel **B**) Electrophoretic profiles of digestive fluids of almond raw (lane 1), autoclaved for 10 min (lane 2) and prehydrated and autoclaved for 10 min (lane 3). M: MW reference standard. Bands submitted to in-gel tryptic digestion for further HPLC-MS/MS analysis, were marked with letters (U1-U6 for undigested sample) and numbers (band 1a and 1b were processed as single band).

Based on the ELISA results, we decided to restrict the number of almond samples to be submitted to gastro-duodenal digestion. Accordingly, only raw almonds (CTRL), almonds autoclaved for 10 min (AC10) and almonds prehydrated/autoclaved for 10 min (H_2_O-AC10) underwent simulated digestion. The most extensive heating applied namely 20 min of autoclaving was excluded because harsher conditions may in general result in detrimental alteration of organoleptic or nutritional properties of almonds. Several papers demonstrated that extensive thermal processing can induce physiochemical alterations of some important constituents such as vitamin, protein, lipids, or bioactive compounds (e.g., polyphenols) often compromising the final nutritional quality of processed food [45,46,47]. In addition, it was experimentally noticed that 20 min autoclaving produced almonds browner in color and an alteration in their appearance and texture compared to 10 min processing. These phenomena were even more enhanced in almonds prehydrated and autoclaved for 20 min, for which a dramatic alteration of the consistency was observed above all. Therefore, since our goal was to find a processing method able to reduce the allergenicity of almond and minimizing, at the same time, the losses in the respective sensory and nutritional quality, we decided to focus for further experiments on 10 min autoclaving treatment, that was found to be a good compromise between its consistency and the decrease in allergenicity. Digestion experiments were accomplished according to a standardized protocol mimicking chewing, gastric and intestinal compartments [29]. A complementary experiment aimed to investigate the composition of the protein fraction in biological fluids, representing the actual amount of proteins solubilized in the digestive fluids and potentially susceptible to enzymatic hydrolysis, was performed on raw almonds. Specifically, raw almond flour was submitted to chew (2 min), gastric (2 h) and duodenal (2 h) phases (simulated by the respective fluids), but no digestive enzymes were added during the procedure. Concerning the simulation of the physiological digestion, raw, autoclaved, and prehydrated/autoclaved almond flour samples were submitted to the original procedure where all the enzymes, specific for each compartment, were added. Total proteins resulting at the end of the two different experiments were analyzed by SDS-PAGE and the respective profiles are reported in Figure 4 (panel A and B). Each relevant protein band was further analyzed by HPLC-MS/MS and identified via bioinformatic database searches using a commercial software (Table 1). A typical protein profile of raw almonds extracted with biological fluids (SSF, SGF and SIF at physiological conditions 37 °C) is reported in Figure 4, lane 1. This protein pool could represent our point “0”, namely the protein profile present in undigested sample extract. Different bands were displayed, specifically above 50 kDa and in the ranges of 37–50 kDa, 20–25 kDa and below 16 kDa. According to LC-MS/MS analysis protein banding above 50 kDa (Panel A, U1) was assigned to (R)-mandelonitrile lyase isoenzyme 2 as well as band named U2 of MW of approximately 50 kDa. Band U3 was attributed to a protein involved in response to water stimulus, namely the abscisic acid response protein. Unexpectedly, prunin was assigned to the bands tagged as U4, U5 and U6 with MW around 20, 15 and 10 kDa, respectively. Moreover, the high intensity of band U6 suggested that the protein was already partially fragmented by passing from SSF into SGF and SIF fluids, although no enzymes were added at this step.

Prunin was recognized as the major water-soluble storage protein in almonds, and it is likely that the drastic pH change occurring from the neutral environment of SSF (pH 7) and acidic compartment of SGF (pH 3) affected prunin stability, resulting in the spontaneous protein hydrolysis (fragments banding below 20 kDa). Such hypothesis appeared consistent with the work authored by Tiwari et al. in 2010, who reported that some denaturation or destruction phenomena of the pruning protein occurred at acidic pH [48]. 

In addition, in Figure 4, panel B, we presented the electrophoretic profiles of raw (lane 1), autoclaved (lane 2) and prehydrated autoclaved (lane 3) almonds submitted to the entire digestion protocol (chew, gastric and intestinal phase with the addition of all digestion enzymes). The more relevant protein bands displayed in raw and autoclaved digested sample were identified by HPLC-MS/MS experiments followed by bioinformatics search, with the respective results listed in Table 1. At a first glance, by comparing the protein profiles shown in panel A and B of Figure 4, we can clearly appreciate the change in almond protein profile after digestion, along with the effect of the treatments tested on protein digestibility. Focusing on digested raw almonds (Figure 4, panel B, lane 1), protein profiles obtained in the beginning and at the end of the simulated gastro-duodenal digestion appeared to be very different. Firstly, an additional band with MW of approximately 50 kDa was displayed in almond digests (panel B, lane 1) along with the protein banding above 50 kDa, already detected in undigested sample (U1 of panel A, lane 1). In both samples (undigested: panel A, lane 1, band U1; and digested: panel B, lane 1, band 1a) this band was attributed to R-mandeonitrile lyase isoenzyme 2, while the additional band detected in digested samples (panel B, lane 1, band 1b) was assigned to one of the digestive enzyme (pancreatic alpha-amylase). In addition, new proteins bands appeared after digestion of raw samples banding around at 25–37 kDa, marked as 3, 4, and 5 (panel B, lane 1). All these bands were attributed to a mixture of digestive enzymes (Table 1). Interestingly, the intense protein bands in the region of 10–20 kDa, visible in undigested sample and attributed to prunin (panel A, lane 1, bands U4, U5 and U6) were missing in the digested samples, suggesting that likely the full degradation upon digestion of this allergenic protein occurred. In the same place, only a smear band was visible (panel B, lane 1, band 6) attributed to trypsin. Autoclaved almond digests (Figure 4, panel B, lane 2) provided protein profiles similar to that of raw samples, apart from one band corresponding to R-mandeonitrile lyase enzyme that disappeared after digestion (see band 1a in lane 1, Figure 4, panel B, corresponding to digested raw almond). Other detectable bands (panel B, lane 2, bands 7–12) referred to digestive enzymes (Table 1). Digestion of prehydrated/autoclaved samples (panel B, lane 3) produced an electrophoretic profile similar to those already observed for autoclaved sample digest. The few bands detected in the gel (panel B, lane 3, bands 13–18) were assigned to digestive enzymes (Table 1). 

Digestibility of almond proteins, specifically prunin (Pru du 6 allergen), was already investigated by other authors. Mandalari et al., in 2014 studied the kinetics of prunin digestion during simulated gastro-duodenal digestion finding that at the end of the process, the only almond protein detectable was a R-oxynitrile lyase isoenzyme 1. Prunin remaining after gastric digestion was broken down after 0.2 min of duodenal digestion, thus no trace of that protein was visible in SDS-PAGE [39]. On the contrary Toomer et al., in 2013 reported that prunin was only partially hydrolyzed by digestive enzymes, preserving the integrity of a protein at 20–22 kDa after pancreatin digestion [38]. Our results agree with the investigation accomplished by Mandalari et al. (2014), showing that no bands corresponding to prunin (comprised in the range 10–20 kDa) were detectable at the end of the gastro-duodenal digestion in raw almond digests, confirming the susceptible behavior of this protein to digestive enzymes. Similar results were obtained also when treated samples, namely autoclaved and prehydrated/autoclaved almonds, underwent digestion, showing that both approaches do not alter the final digestibility of almond proteins, specifically in the case of prunin that is the major almond allergen. 

It is worth noting that the present study does not take into account the potential effect of polyphenols compounds in the modulation of the final digestibility of almond proteins, since peeled almond kernels were used in all the experiments performed. Polyphenols compounds were found rich in almond skin and they were frequently related to the several health benefits associated with almond consumption [36,49,50]. Also, several papers demonstrated the ability of these compounds to bind proteins (recently reviewed by Jacobelk et al., 2015 and Ozdal et al., 2013 [51,52]. Consequently, some alterations in the amino acid availability were observed, along with the occurrence of denaturation phenomena and the reduction of the protein digestibility. About this last issue, Mandalari et al., demonstrated in 2016 that in full-fat milk enriched with almond skins, the bioaccessibility of polyphenols during simulated human digestion was deeply reduced, likely due to protein-polyhenols interactions [37]. By eliminating skins from almond kernels as detailed in the present work, we deliberately eliminated any source of interferences (polyphenols or others) in presence of skin, providing therefore more insights on the effect of the solely heat treatment to assess allergen stability or protein digestibility. 

Finally, to have complementary information about the digestibility of almond proteins, raw and treated almond samples, collected at the end of the duodenal phase, were loaded on Size Exclusion Chromatography (SEC) cartridges (6 kDa cut-off) and the lower MW fraction (lower than 6 kDa) was directly analyzed by HPLC-MS/MS. In Table 2 are summarized the allergenic proteins whose fragments were identified in the low MW range of the duodenal digests. In raw almond digests, most of peptides were assigned to Pru du 6. The presence of this allergen in the <6 kDa fraction underlined the high degree of fragmentation occurred to this molecule upon digestion, and thus its high susceptibility to GI enzymes. In addition, peptides assigned to other allergenic proteins were identified in the low MW fraction of digested raw almonds, such as Pru du 3, Pru du 4, Pru du 5 and Pru 2S Albumin, which probably were not highlighted in the electrophoretic pattern because of the low MW of intact proteins (9, 14, 11 and 12 kDa, respectively). Pru du Allergenic Protein (AP) (also named Pru du γ-conglutin, original MW 45 kDa) was also identified in the low MW protein fraction of raw almonds, confirming the susceptibility of this allergen to digestive enzymes. Proteins identified in this fraction corresponding to autoclaved and prehydrated/autoclaved digested almonds were similar to that reported in raw almond digests, although a different number of peptides was found for each allergen (Table 2). Interestingly, in comparison with the raw almond digests, the number of unique peptides attributed to Pru du 6 remained stable in autoclaved samples, but increased in prehydrated/autoclaved samples, suggesting that the combined effect of both and the presence of digestive enzymes lead to a higher fragmentation of the protein. A different trend was found for Pru du 3, Pru du 4, Pru du 5 and Pru du AP where total peptides number appeared to decrease when passing from raw to treated almond digests (Table 2). It should be hypothesized that heat/pressure effect, combined with the proteolytic activity of digestive enzymes, promoted the extensive degradation of peptides down to fragment lower that 5 AA in length, which were missed by the software-based identification, resulting in a reduced number of total detected peptides. As for Pru du 2S albumin, no difference in peptide number was displayed between raw and processed samples, likely due to a high resistance of the protein to the investigated treatments. In general, these results complemented the information provided by Table 1, supporting the previous observation made on the electrophoretic profiles of undigested and digested almonds. As discussed above, the content of prunin (Pru du 6), particularly abundant in the undigested electrophoretic profile, disappeared upon gastro-duodenal digestion and as a confirmation, in this low MW fraction, a large number of peptides belonging to this protein was detected. Similar results were obtained in almonds thermally/pressure treated (with previous hydration or not) demonstrating that these technological approaches did not impair the final digestibility of almond allergens. On the contrary, in some cases, they improved the protein fragmentation, with probable potential influence in the final allergenicity. To the best of our knowledge, this is the first work investigating the digestibility of almond proteins after thermal/pressure treatment. The digestibility of blanched almond proteins was studied by Mandalari et al. in 2014, and, similarly to the results reported here, no significant differences were observed between the kinetics of digestion of natural and blanched almond proteins [39].

#### 3.4.2. Assessment of Residual Immunoreactivity of Thermally/Pressure Treated Almonds Submitted to In Vitro Digested 

As known, food processing may induce physical or chemical modifications that deeply affect the final structure/ conformation of a proteins, often altering their final digestibility, that is strictly linked with their potential immunoreactivity. In light of this, the final section of our work was aimed at investigating the immunoreactive potential of the digested almond proteins raw and treated with autoclave (with or without hydration), scouting for full-length linear epitopes encrypted by the identified resistant peptide sequences, by means of bioinformatics tools. All peptides contained in the low MW fraction of the duodenal digest were taken into consideration. The IEDB database was screened to match detected peptides and almond linear epitopes recognized for the *Homo sapiens* host. For this investigation, only peptides with sequence length >9 AA were considered, in accordance with the EFSA guidelines set up to test the allergenicity of in vitro digested proteins [53]. No intact epitopes reported in IEDB database were found to match with the peptides included in low MW protein fraction of digested almonds untreated and treated with the two different approaches. In Table S1 of supplementary data, peptide sequences searched in IEDB database along with the belonging proteins were shown. Despite arisen from allergenic proteins, no peptides were found to preserve an intact epitopic sequence. Although results appeared be very promising, they need to be further confirmed by specific immunological analysis (e.g., immunoblotting with patients’ sera allergic to almonds). Indeed, this kind of approach substantially based on bioinformatics analysis could present limitations due to the restricted number of almond epitopes sequenced and deposited in IEDB database. Anyway, our preliminary results are in line with those reported by Mandalari et al., 2014 on the residual immunoreactivity of natural almonds digested by a dynamic digestion protocol. They found that dot blot signals produced by probing materials with rabbit pAb and two murine mABs (mAb 2A3 specific for a linear epitope of prunin and mAb 4C10 specific for a conformational almond epitope) were significantly decreased with respect to undigested sample, and this trend was confirmed by ELISA assay. On the contrary, the same authors stated that when incorporated into processed food (Victoria sponge cake and chocolate mousse), the digestibility of almond proteins resulted slowed down by passing from gastric to duodenal compartments, with consequence persistence of some immunoreactivity at the end of digestion, mainly visible in chocolate mousse sample [39]. To the best of our knowledge, the effect of autoclaving treatment on the residual immunological potential of almonds was never investigated before by tracking the fate of the allergenic protein upon simulated gastro-duodenal digestion, therefore our promising results could open the way for further analysis in this direction. Moreover, considering the importance of the food matrix in the modulation of the digestibility, and consequently the allergenicity, of almond proteins, further studies aimed at investigating the immunoreactivity of thermally/pressure treated almonds-based composite food commodities should be very interesting from a toxicological point of view. 

## 4. Conclusions

In this study, the combination of heat/pressure treatments (autoclave), performed at 134 °C, 2 atm, was investigated for its potential to reduce almond allergenicity. Almond seeds were submitted to autoclave treatment, preceded or not by a hydration step, and the respective protein recovery was assessed by Bradford assay, along with the evaluation of their immunoreactivity by ELISA tests. Finally, any change induced by autoclaving treatments on almond proteins digestibility was evaluated by in vitro digestion experiments, and the persistence of digestive immunoreactive peptides was assessed by bioinformatic analysis. Our results showed that the synergist effect of heat and pressure resulted in a visible alteration of almond proteins stability, inducing a final reduction of the total immunoreactivity in almonds. In particular, hydration before autoclaving proved to increase the efficacy of the thermal/pressure treatment, contributing to the disappearance of the main allergenic protein bands and altering significantly the final immunoreactivity. Furthermore, by submitting the treated almonds to in vitro digestion experiments a full degradation of the main almond allergens took place and the residual immunoreactivity estimated by bioinformatics analysis turn out to be negligible. This demonstrated that the autoclave-based treatment may induce a drastic reduction of the overall allergenicity in almonds, especially when preceded by incubation in water. In perspective, this represents a first step towards the development of effective processing techniques to reduce tree nut immunoreactivity and may prove useful in the development of strategies for food tolerance induction and/or to establish threshold levels of sensitization/elicitation for hypoallergenic foods. However, further investigations are required, mainly employing immunological techniques, to evaluate any loss of residual allergenicity of processed almonds on allergic patients.

## Figures and Tables

**Table 1:** List of proteins identified by HPLC-MS/MS analysis followed by software data processing of selected bands in-gel digested referred to raw almond samples undigested (CTRL-NE) and digested (CTRL-GI) along with digested almond autoclaved (AC10-GI) and prehydrated autoclaved (H_2_O-AC10-GI). All the relevant software parameters were also included.

Sample	Band	Accession Number	Type of Protein (Organism)	Allergen	Coverage (%)	Unique Peptides	Score
CTRL-NE	U1	Q945K2	(R)-mandelonitrile lyase 2 (*Prunus dulcis* (Mill.) D. A. Webb)		30.37	18 (18)	30.34
	U2	Q945K2	(R)-mandelonitrile lyase 2 (*Prunus dulcis* (Mill.) D. A. Webb)		11.55	7 (7)	0
	U3	Q9SW89	Abscisic acid response protein (*Prunus dulcis* (Mill.) D. A. Webb)		43.07	8 (8)	19.35
	U4	Q43607	Prunin (*Prunus dulcis* (Mill.) D. A. Webb)	Pru du 6	12.16	8 (8)	1.65
	U5	Q43607	Prunin (*Prunus dulcis* (Mill.) D. A. Webb)	Pru du 6	8.53	6 (1)	8.59
		E3SH28	Prunin 1 (*Prunus dulcis* (Mill.) D. A. Webb)	Pru du 6, Pru du 6.0101	8.17	6 (1)	6.94
	U6	Q43607	Prunin (*Prunus dulcis* (Mill.) D. A. Webb)	Pru du 6	16.33	11 (2)	21.8
		E3SH28	Prunin 1 (*Prunus dulcis* (Mill.) D. A. Webb)	Pru du 6, Pru du 6.0101	15.25	10 (1)	16.56
		A7Y7K3	Putative lipid-transfer protein (*Prunus dulcis* (Mill.) D. A. Webb)		40.91	3 (3)	9.77
		E3SH29	Prunin 2 (Fragment) (*Prunus dulcis* (Mill.) D. A. Webb)	Pru du 6, Pru du 6.0201	13.10	7 (7)	4.43
CTRL-GI	1	Q945K2	(R)-mandelonitrile lyase 2 (*Prunus dulcis* (Mill.) D. A. Webb)		36.41	31 (31)	17.64
		P06278	Alpha-amylase *(Bacillus licheniformis*)		9.96	10 (10)	1.77
	2	P00690	Pancreatic alpha-amylase (*Sus scrofa* Linnaeus)		70.45	55 (28)	100.16
		P04745	Alpha-amylase (*Homo sapiens* Linnaeus)		33.27	26 (4)	58.45
		Q945K2	(R)-mandelonitrile lyase 2 (*Prunus dulcis* (Mill.) D. A. Webb)		21.31	12 (12)	1.83
	3	P00690	Pancreatic alpha-amylase (*Sus scrofa* Linnaeus)		15.66	8 (8)	0
	4	P00690	Pancreatic alpha-amylase (*Sus scrofa* Linnaeus)		25.83	21 (20)	10.21
		Q7M3E1	Chymotrypsin-C (*Bos taurus* Linnaeus)		7.09	3 (2)	3.18
	6	P00761	Trypsin (*Sus scrofa* Linnaeus)		22.08	5 (4)	3.73
AC10-GI	7	P06278	Alpha-amylase (*Bacillus licheniformis*)		3.32	3 (3)	2.05
	8	P00690	Pancreatic alpha-amylase (*Sus scrofa* Linnaeus)		60.86	47 (23)	80.76
	9	P00690	Pancreatic alpha-amylase (*Sus scrofa* Linnaeus)		33.86	24 (23)	18.86
	10	P00690	Pancreatic alpha-amylase (*Sus scrofa* Linnaeus)		19.37	13 (13)	10.92
	11	P00766	Chymotrypsinogen A (*Bos taurus*)		15.10	5 (4)	3.53
	12	P00761	Trypsin (*Sus scrofa* Linnaeus)		18.61	5 (4)	6.36
		P00690	Pancreatic alpha-amylase (*Sus scrofa* Linnaeus)		13.50	10 (9)	5.46
H_2_O-AC10-GI	14	P00690	Pancreatic alpha-amylase (*Sus scrofa* Linnaeus)		50.68	44 (22)	58.65
	15	P00690	Pancreatic alpha-amylase (*Sus scrofa* Linnaeus)		30.14	20 (19)	9.4
	16	P00690	Pancreatic alpha-amylase (*Sus scrofa* Linnaeus)		19.77	14 (14)	9.04
		Q7M3E1	Chymotrypsin-C (*Bos taurus* Linnaeus)		7.09	3 (3)	5.08
	17	P00766	Chymotrypsinogen A (*Bos taurus* Linnaeus)		11.02	3 (2)	9.49
	18	P00761	Trypsin (*Sus scrofa* Linnaeus)		39.83	9 (9)	11.92
		P00690	Pancreatic alpha-amylase (*Sus scrofa* Linnaeus)		12.72	7 (7)	6.03

**Table 2:** List of proteins identified by HPLC-MS/MS analysis followed by bioinformatic search via commercial software of low molecular weight fraction (<6 kDa) isolated from raw almond digest of (CTRL-GI), autoclaved (AC10-GI) and prehydrated autoclaved almond (H_2_O-AC-GI) along with the relevant parameters provided by software.

Sample	Accession Number	Type of Protein (Organism)	Allergen	Coverage (%)	Unique Peptides	Score
CTRL-GI	C0L0I5	Non-specific lipid-transfer protein (*Prunus dulcis* (Mill.) D. A. Webb)	Pru du 3, Pru du 3.0101	40.65	11 (10)	4.11
	P82944	Seed allergenic protein 1 (Fragments) (*Prunus dulcis* (Mill.) D. A. Webb)	Pru du 2S Albumin	60.71	5 (4)	10.21
	Q43608	Pru2 protein (Fragment) (*Prunus dulcis* (Mill.) D. A. Webb)	Pru du 6	71.83	195 (7)	297.12
	E3SH28	Prunin 1 (*Prunus dulcis* (Mill.) D. A. Webb)	Pru du 6, Pru du 6.0101	80.76	323 (4)	672.17
	Q8GSL5	Profilin (*Prunus dulcis* (Mill.) D. A. Webb)	Pru du 4	40.46	17 (17)	9.7
	P82952	Seed allergenic protein 2 (Fragment) (*Prunus dulcis* (Mill.) D. A. Webb)	Pru du AP	48.00	15 (15)	39.02
	Q43607	Prunin (*Prunus dulcis* (Mill.) D. A. Webb)	Pru du 6	81.85	335 (14)	697.42
	Q8H2B9	60S acidic ribosomal protein (*Prunus dulcis* (Mill.) D. A. Webb)	Pru du 5, Pru du 5.0101	37.17	23 (22)	9.42
AC10-GI	E3SH29	Prunin 2 (Fragment) (*Prunus dulcis* (Mill.) D. A. Webb)	Pru du 6, Pru du 6.0201	68.65	213 (4)	363.33
	C0L0I5	Non-specific lipid-transfer protein (*Prunus dulcis* (Mill.) D. A. Webb)	Pru du 3, Pru du 3.0101	32.52	9 (9)	2.57
	P82944	Seed allergenic protein 1 (Fragments) (*Prunus dulcis* (Mill.) D. A. Webb)	Pru du 2S Albumin	60.71	5 (4)	11.76
	Q43608	Pru2 protein (Fragment) (*Prunus dulcis* (Mill.) D. A. Webb)	Pru du 6	69.05	209 (6)	359.74
	E3SH28	Prunin 1 (*Prunus dulcis* (Mill.) D. A. Webb)	Pru du 6, Pru du 6.0101	78.40	292 (4)	700.81
	Q8GSL5	Profilin (*Prunus dulcis* (Mill.) D. A. Webb)	Pru du 4	30.53	13 (13)	4.48
	P82952	Seed allergenic protein 2 (Fragment) (*Prunus dulcis* (Mill.) D. A. Webb)	Pru du AP	48.00	10 (10)	17.94
	Q43607	Prunin (*Prunus dulcis* (Mill.) D. A. Webb)	Pru du 6	78.77	300 (10)	712.31
	Q8H2B9	60S acidic ribosomal protein (*Prunus dulcis* (Mill.) D. A. Webb)	Pru du 5, Pru du 5.0101	37.17	13 (13)	5.05
H_2_O-AC10-GI	E3SH29	Prunin 2 (Fragment) (*Prunus dulcis* (Mill.) D. A. Webb)	Pru du 6, Pru du 6.0201	78.97	272 (16)	630.16
	C0L0I5	Non-specific lipid-transfer protein (*Prunus dulcis* (Mill.) D. A. Webb)	Pru du 3, Pru du 3.0101	27.64	7 (6)	1.43
	P82944	Seed allergenic protein 1 (Fragments) (*Prunus dulcis* (Mill.) D. A. Webb)	Pru du 2S Albumin	78.57	6 (5)	8.77
	Q43608	Pru2 protein (Fragment) (*Prunus dulcis* (Mill.) D. A. Webb)	Pru du 6	77.58	261 (10)	592.97
	E3SH28	Prunin 1 (*Prunus dulcis* (Mill.) D. A. Webb)	Pru du 6, Pru du 6.0101	83.30	321 (8)	941.16
	Q8GSL5	Profilin (*Prunus dulcis* (Mill.) D. A. Webb)	Pru du 4	21.37	10 (10)	8.1
	P82952	Seed allergenic protein 2 (Fragment) (*Prunus dulcis* (Mill.) D. A. Webb)	Pru du AP	48.00	9 (9)	21.22
	Q43607	Prunin (*Prunus dulcis* (Mill.) D. A. Webb)	Pru du 6	82.94	320 (5)	954.46
	Q8H2B9	60S acidic ribosomal protein (*Prunus dulcis* (Mill.) D. A. Webb)	Pru du 5, Pru du 5.0101	37.17	16 (16)	8.63

## Supplementary Materials

The following are available online at http://www.mdpi.com/2072-6643/10/11/1679/s1, Table S1: List of <6 kDa peptides arisen from GI simulated digestion of raw (CTRL-GI), autoclaved (AC10) and prehydrated/autoclaved almonds (H2O-AC10) searched in IEDB database for scouting linear epitope sequences.

## References

[1] Costa J., Mafra I., Carrapatoso I., Oliveira M.B.P.P. (2012). Almond allergens: Molecular characterization, detection, and clinical relevance. J. Agric. Food Chem..

[2] Bolling B.W., Chen C.Y.O., McKay D.L., Blumberg J.B. (2011). Tree nut phytochemicals: Composition, antioxidant capacity, bioactivity, impact factors. A systematic review of almonds, Brazils, cashews, hazelnuts, macadamias, pecans, pine nuts, pistachios and walnuts. Nutr. Res. Rev..

[3] Yada S., Lapsley K., Huang G. (2011). A review of composition studies of cultivated almonds: Macronutrients and micronutrients. J. Food Compos. Anal..

[4] Fernández-Cuesta A., Kodad O., Velasco L. (2012). Phytosterol variability in almond germplasm. J. Am. Soc. Hortic. Sci..

[5] FAOSTAT Food and Agriculture Organization of the United Nations. http://www.fao.org/home/en/.

[6] Elizur A., Appel M.Y., Nachshon L., Levy M.B., Epstein-Rigbi N., Golobov K., Goldberg M.R. (2018). NUT Co Reactivity—Acquiring Knowledge for Elimination Recommendations (NUT CRACKER) study. Allergy Eur. J. Allergy Clin. Immunol..

[7] McWilliam V., Koplin J., Lodge C., Tang M., Dharmage S., Allen K. (2015). The Prevalence of Tree Nut Allergy: A Systematic Review. Curr. Allergy Asthma Rep..

[8] Allergen Nomenclature, Allergen Nomenclature IUIS Allergen Nomenclature Sub-Committee. http://www.allergen.org.

[9] Roux K.H., Teuber S.S., Robotham J.M., Sathe S.K. (2001). Detection and stability of the major almond allergen in foods. J. Agric. Food Chem..

[10] Sathe S.K., Wolf W.J., Roux K.H., Teuber S.S., Venkatachalam M., Sze-Tao K.W.C. (2002). Biochemical characterization of amandin, the major storage protein in almond (*Prunus Dulcis* L.). J. Agric. Food Chem..

[11] Garcia-Mas J., Messeguer R., Arús P., Puigdomènech P. (1995). Molecular characterization of cDNAs corresponding to genes expressed during almond (*Prunus amygdalus* Batsch) seed development. Plant Mol. Biol..

[12] Albillos S.M., Jin T., Howard A., Zhang Y., Kothary M.H., Fu T.J. (2008). Purification, crystallization and preliminary X-ray characterization of prunin-1, a major component of the almond (*Prunus dulcis*) allergen amandin. J. Agric. Food Chem..

[13] Alasalvar C., Shahidi F. (2008). Tree Nuts: Composition, Phytochemicals, and Health Effects.

[14] EU Regulation No. (2011). 1169/2011 of the European Parliament and of the Council of 25 October 2011 on the provision of food information to consumers. Off. J. Eur. Union.

[15] Monaci L., De Angelis E., Montemurro N., Pilolli R. (2018). Comprehensive overview and recent advances in proteomics MS based methods for food allergens analysis. TrAC Trends Anal. Chem..

[16] Andjelković U., Gavrović-Jankulović M., Martinović T., Josić D. (2017). Omics methods as a tool for investigation of food allergies. TrAC Trends Anal. Chem..

[17] Pilolli R., De Angelis E., Monaci L. (2017). Streamlining the analytical workflow for multiplex MS/MS allergen detection in processed foods. Food Chem..

[18] Pilolli R., De Angelis E., Monaci L. (2018). In house validation of a high resolution mass spectrometry Orbitrap-based method for multiple allergen detection in a processed model food. Anal. Bioanal. Chem..

[19] Rahaman T., Vasiljevic T., Ramchandran L. (2016). Effect of processing on conformational changes of food proteins related to allergenicity. Trends Food Sci. Technol..

[20] Su M., Liu C., Roux K.H., Gradziel T.M., Sathe S.K. (2017). Effects of processing and storage on almond (*Prunus dulcis* L.) amandin immunoreactivity. Food Res. Int..

[21] Venkatachalam M., Teuber S.S., Roux K.H., Sathe S.K. (2002). Effects of roasting, blanching, autoclaving, and microwave heating on antigenicity of almond (*Prunus dulcis* L.) proteins. J. Agric. Food Chem..

[22] Zhang Y., Zhang J., Sheng W., Wang S., Fu T.J. (2016). Effects of heat and high-pressure treatments on the solubility and immunoreactivity of almond proteins. Food Chem..

[23] Shriver S.K., Yang W.W. (2011). Thermal and Nonthermal Methods for Food Allergen Control. Food Eng. Rev..

[24] Albillos S.M., Menhart N., Fu T.J. (2009). Structural stability of amandin, a major allergen from almond (*Prunus dulcis*), and its acidic and basic polypeptides. J. Agric. Food Chem..

[25] Su M., Venkatachalam M., Teuber S.S., Roux K.H., Sathe S.K. (2004). Impact of γ-irradiation and thermal processing on the antigenicity of almond, cashew nut and walnut proteins. J. Sci. Food Agric..

[26] Li Y., Yang W., Chung S.Y., Chen H., Ye M., Teixeira A.A., Gregory J.F., Welt B.A., Shriver S. (2013). Effect of Pulsed Ultraviolet Light and High Hydrostatic Pressure on the Antigenicity of Almond Protein Extracts. Food Bioprocess Technol..

[27] Dhakal S., Liu C., Zhang Y., Roux K.H., Sathe S.K., Balasubramaniam V.M. (2014). Effect of high pressure processing on the immunoreactivity of almond milk. Food Res. Int..

[28] Bøgh K.L., Madsen C.B. (2016). Food Allergens: Is There a Correlation between Stability to Digestion and Allergenicity?. Crit. Rev. Food Sci. Nutr..

[29] Minekus M., Alminger M., Alvito P., Ballance S., Bohn T., Bourlieu C., Carrière F., Boutrou R., Corredig M., Dupont D. (2014). A standardised static in vitro digestion method suitable for food-an international consensus. Food Funct..

[30] (2010). EFSA Panel on Genetically Modified Organisms (GMO), Draft Scientific Opinion on the assessment of allergenicity of GM plants and microorganisms and derived food and feed. EFSA J..

[31] Mandalari G., Faulks R.M., Rich G.T., Lo Turco V., Picout D.R., Lo Curto R.B., Bisignano G., Dugo P., Dugo G., Waldron K.W. (2008). Release of protein, lipid, and vitamin E from almond seeds during digestion. J. Agric. Food Chem..

[32] Mandalari G., Grundy M.M.L., Grassby T., Parker M.L., Cross K.L., Chessa S., Bisignano C., Barreca D., Bellocco E., Laganà G. (2014). The effects of processing and mastication on almond lipid bioaccessibility using novel methods of in vitro digestion modelling and micro-structural analysis. Br. J. Nutr..

[33] Mandalari G., Parker M.L., Grundy M.M.L., Grassby T., Smeriglio A., Bisignano C., Raciti R., Trombetta D., Baer D.J., Wilde P.J. (2018). Understanding the effect of particle size and processing on almond lipid bioaccessibility through microstructural analysis: From mastication to faecal collection. Nutrients.

[34] Grassby T., Mandalari G., Grundy M.M.L., Edwards C.H., Bisignano C., Trombetta D., Smeriglio A., Chessa S., Ray S., Sanderson J. (2017). In vitro and in vivo modeling of lipid bioaccessibility and digestion from almond muffins: The importance of the cell-wall barrier mechanism. J. Funct. Foods.

[35] Grassby T., Picout D.R., Mandalari G., Faulks R.M., Kendall C.W.C., Rich G.T., Wickham M.S.J., Lapsley K., Ellis P.R. (2014). Modelling of nutrient bioaccessibility in almond seeds based on the fracture properties of their cell walls. Food Funct..

[36] Mandalari G., Tomaino A., Rich G.T., Lo Curto R., Arcoraci T., Martorana M., Bisignano C., Saija A., Parker M.L., Waldron K.W. (2010). Polyphenol and nutrient release from skin of almonds during simulated human digestion. Food Chem..

[37] Mandalari G., Vardakou M., Faulks R., Bisignano C., Martorana M., Smeriglio A., Trombetta D. (2016). Food matrix effects of polyphenol bioaccessibility from almond skin during simulated human digestion. Nutrients.

[38] Toomer O.T., Do A., Pereira M., Williams K. (2013). Effect of simulated gastric and intestinal digestion on temporal stability and immunoreactivity of peanut, almond, and pine nut protein allergens. J. Agric. Food Chem..

[39] Mandalari G., Rigby N.M., Bisignano C., Lo Curto R.B., Mulholland F., Su M., Venkatachalam M., Robotham J.M., Willison L.N., Lapsley K. (2014). Effect of food matrix and processing on release of almond protein during simulated digestion. LWT Food Sci. Technol..

[40] Bavaro S.L., Di Stasio L., Mamone G., De Angelis E., Nocerino R., Canani R.B., Logrieco A.F., Montemurro N., Monaci L. (2018). Effect of thermal/pressure processing and simulated human digestion on the immunoreactivity of extractable peanut allergens. Food Res. Int..

[41] De Angelis E., Pilolli R., Bavaro S.L., Monaci L. (2017). Insight into the gastro-duodenal digestion resistance of soybean proteins and potential implications for residual immunogenicity. Food Funct..

[42] Masthoff L.J., Hoff R., Verhoeckx K.C.M., van Os-Medendorp H., Michelsen-Huisman A., Baumert J.L., Pasmans S.G., Meijer Y., Knulst A.C. (2013). A systematic review of the effect of thermal processing on the allergenicity of tree nuts. Allergy.

[43] Cabanillas B., Cuadrado C., Rodriguez J., Hart J., Burbano C., Crespo J.F., Novak N. (2015). Potential changes in the allergenicity of three forms of peanut after thermal processing. Food Chem..

[44] Cabanillas B., Maleki S.J., Rodríguez J., Cheng H., Teuber S.S., Wallowitz M.L., Muzquiz M., Pedrosa M.M., Linacero R., Burbano C. (2014). Allergenic properties and differential response of walnut subjected to processing treatments. Food Chem..

[45] Awuah G.B., Ramaswamy H.S., Economides A. (2007). Thermal processing and quality: Principles and overview. Chem. Eng. Process. Process Intensif..

[46] Ioannou I., Hafsa I., Hamdi S., Charbonnel C., Ghoul M. (2012). Review of the effects of food processing and formulation on flavonol and anthocyanin behaviour. J. Food Eng..

[47] Lešková E., Kubíková J., Kováčiková E., Košická M., Porubská J., Holčíková K. (2006). Vitamin losses: Retention during heat treatment and continual changes expressed by mathematical models. J. Food Compos. Anal..

[48] Tiwari R.S., Venkatachalam M., Sharma G.M., Su M., Roux K.H., Sathe S.K. (2010). Effect of food matrix on amandin, almond (*Prunus dulcis* L.) major protein, immunorecognition and recovery. LWT Food Sci. Technol..

[49] Mandalari G., Genovese T., Bisignano C., Mazzon E., Wickham M.S.J., Di Paola R., Bisignano G., Cuzzocrea S. (2011). Neuroprotective effects of almond skins in experimental spinal cord injury. Clin. Nutr..

[50] Mandalari G., Bisignano C., Genovese T., Mazzon E., Wickham M.S.J., Paterniti I., Cuzzocrea S. (2011). Natural almond skin reduced oxidative stress and inflammation in an experimental model of inflammatory bowel disease. Int. Immunopharmacol..

[51] Ozdal T., Capanoglu E., Altay F. (2013). A review on protein-phenolic interactions and associated changes. Food Res. Int..

[52] Jakobek L. (2015). Interactions of polyphenols with carbohydrates, lipids and proteins. Food Chem..

[53] Naegeli H., Birch A.N., Casacuberta J., De Schrijver A., Gralak M.A., Guerche P., Jones H., Manachini B., Messéan A., Nielsen E.E. (2017). EFSA Panel on Genetically Modified Organisms (GMO). EFSA J..

