# Supplementary material for: Heat and Pressure Treatments on Almond Protein Stability and Change in Immunoreactivity after Simulated Human Digestion
Source: Nutrients. 2018 Nov 5;10(11):1679. doi: 10.3390/nu10111679 (PMC6265937; doi:10.3390/nu10111679)
Supplement: Supplementary file 1 [file nutrients-10-01679-s001.pdf]

Supplementary table. List of < 6 kDa peptides arisen from GI simulated digestion of raw (CTRL-GI), autoclaved (AC10) and prehydrated/autoclaved almonds (H_2O-AC10) searched in IEDB database for scouting linear epitope sequences.

Sample	Sequence	Protein Accession number	Allergen	Epitope ID	Epitopic sequence	Rif
CTRL-GI	HVSSDHNQLDQNPR	E3SH28; Q43607	Pru du 6	-	-	-
	FHVSSDHNQLDQNPR	E3SH28; Q43607	Pru du 6	-	-	-
	HVSSDHNQLDQNP	E3SH28; Q43607	Pru du 6	-	-	-
	TKSQTHVPIRP	P82952	Pru du AP	-	-	-
	TKSQTHVPIRPN	P82952	Pru du AP	-	-	-
	GEQGRPGQHQ	E3SH28; Q43607	Pru du 6	-	-	-
	VSSDHNQLDQNPR	E3SH28; Q43607	Pru du 6	-	-	-
	QEQQQGQQGRPQ	E3SH28; Q43607	Pru du 6	-	-	-
	EQQQGQQGRPQ	E3SH28; Q43607	Pru du 6	-	-	-
	QQGEQGRPGQH	E3SH28; Q43607	Pru du 6	-	-	-
	FHVSSDHNQLDQNP	E3SH28; Q43607	Pru du 6	-	-	-
	QQGEQGRPGQHQQP	E3SH28; Q43607	Pru du 6	-	-	-
	QQGEQGRPGQHQQPF	E3SH28; Q43607	Pru du 6	-	-	-
	QGQDDNRNEI	E3SH29; Q43608	Pru du 6	-	-	-
	GNLDFVQPPR	E3SH28; Q43607	Pru du 6	-	-	-
	RGNLDFVQPPR	E3SH28; Q43607	Pru du 6	-	-	-
	QQQGQQGRPQ	E3SH28; Q43607	Pru du 6	-	-	-
	QQGEQGRPGQ	E3SH28; Q43607	Pru du 6	-	-	-
	RQQQQQGEQG	E3SH28; Q43607	Pru du 6	-	-	-
	QQQEEGRQQEQ	E3SH28; Q43607	Pru du 6	-	-	-
	QAREPDNRIQ	E3SH28; Q43607	Pru du 6	-	-	-
	AGNPQDEFNPQ	E3SH29; Q43608; S5LMS1	Pru du 6	-	-	-
	QQQQQGEQGRQQGQ	E3SH28; Q43607	Pru du 6	-	-	-
	GEQGRPGQHQQPF	E3SH28; Q43607	Pru du 6	-	-	-
	ERQQQQQGEQG	E3SH28; Q43607	Pru du 6	-	-	-
	QQEQQGNGNNV	Q43607	Pru du 6	-	-	-

GNLDFVQPPRG	E3SH28; Q43607	Pru du 6	-	-	-
QQGQQEQQQE	E3SH28; Q43607	Pru du 6	-	-	-
GQDDNRNEIV	E3SH29; Q43608	Pru du 6	-	-	-
REGDIIALPA	E3SH29; Q43608; S5LMS1	Pru du 6	-	-	-
NENGDAILDQE	E3SH28; Q43607	Pru du 6	-	-	-
QQQQGEQGRQQGQ	E3SH28; Q43607	Pru du 6	-	-	-
RLKENIGNPE	E3SH28; Q43607	Pru du 6	-	-	-
RIQAEAGQIET	E3SH28; Q43607	Pru du 6	-	-	-
IVPQNHGVIQQ	E3SH28; Q43607	Pru du 6	-	-	-
TESWNPSDPQ	E3SH29; Q43608; S5LMS1	Pru du 6	-	-	-
QQEQQQGQQGRPQ	E3SH28; Q43607	Pru du 6	-	-	-
KSQTHVPIRPN	P82952	Pru du AP	-	-	-
NPQDEFNPQQQGR	E3SH29; Q43608	Pru du 6	-	-	-
RLKENIGNPERAD	E3SH28; Q43607	Pru du 6	-	-	-
EPDNRIQAEAGQIET	E3SH28; Q43607	Pru du 6	-	-	-
GEQGRPGQHQQP	E3SH28; Q43607	Pru du 6	-	-	-
SQTHVPIRPN	P82952	Pru du AP	-	-	-
GGAVAVAAPGAGAGAAAPAA	Q8H2B9	Pru du 5	-	-	-
NENGDAILDQ	E3SH28; Q43607	Pru du 6	-	-	-
IVPQNHGVIQ	E3SH28; Q43607	Pru du 6	-	-	-
LDLNNDQNQLDQVPR	E3SH29; Q43608	Pru du 6	-	-	-
QEQQGNGNNV	Q43607	Pru du 6	-	-	-
RQQEQQGNGNNV	Q43607	Pru du 6	-	-	-
QGGGGQDNGVEET	E3SH29; Q43608	Pru du 6	-	-	-
VIQQAGNQGFE	E3SH28; Q43607	Pru du 6	-	-	-
IQAEAGQIET	E3SH28; Q43607	Pru du 6	-	-	-
RGNLDFVQPP	E3SH28; Q43607	Pru du 6	-	-	-
QVVNENGDPI	E3SH29; Q43608	Pru du 6	-	-	-
REPDNRIQAEAGQIET	E3SH28; Q43607	Pru du 6	-	-	-
NLQGQNDNRNQ	E3SH28; Q43607	Pru du 6	-	-	-
EEGRQQEQQQGQQGRPQ	E3SH28; Q43607	Pru du 6	-	-	-
QQQEEGRQQE	E3SH28; Q43607	Pru du 6	-	-	-

	LRALPDEVLA	E3SH28; Q43607	Pru du 6	-	-	-
	LRLKENIGNPE	E3SH28; Q43607	Pru du 6	-	-	-
	QQEEGRQQEQ	E3SH28; Q43607	Pru du 6	-	-	-
	LAGNPQDEFN	E3SH29; Q43608; S5LMS1	Pru du 6	-	-	-
	AGAGAAAPAAAEPK	Q8H2B9	Pru du 5	-	-	-
	LAGNPENEFN	E3SH28; Q43607	Pru du 6	-	-	-
	NENGDAILDQEVQQG	E3SH28; Q43607	Pru du 6	-	-	-
	QLSPQNQCQLNQL	E3SH28; Q43607	Pru du 6	-	-	-
	SGGGAVAVAAPGAGA	Q8H2B9	Pru du 5	-	-	-
	TIALSSSQQR	E3SH28; Q43607	Pru du 6	-	-	-
	SRSAGGRGDQ	E3SH29; Q43608	Pru du 6	-	-	-
	AGLAAGLPGK	C0L0I5	Pru du 3	-	-	-
	QQEQQGNGNN	Q43607	Pru du 6	-	-	-
	TFEESQQSSQ	E3SH28; Q43607	Pru du 6	-	-	-
	LQGQNDNRNQIIQ	Q43607	Pru du 6	-	-	-
	QQQQQQQGQQGNGNN	E3SH29; Q43608	Pru du 6	-	-	-
	RQQEQQGNGN	Q43607	Pru du 6	-	-	-
	VAVAAPGAGAGAAAP	Q8H2B9	Pru du 5	-	-	-
	AQALNVNPETAR	E3SH29; Q43608	Pru du 6	-	-	-
	RLSQNIGDPS	E3SH29; Q43608	Pru du 6	-	-	-
	STLNSHNLPI	E3SH28; Q43607	Pru du 6	-	-	-
	SGGGAVAVAAPG	Q8H2B9	Pru du 5	-	-	-
	IREGDIIALPA	E3SH29; Q43608; S5LMS1	Pru du 6	-	-	-
	PSGGGAVAVAA	Q8H2B9	Pru du 5	-	-	-
	RALPDEVLAN	E3SH28; Q43607	Pru du 6	-	-	-
	AILDQEVQQG	E3SH28; Q43607	Pru du 6	-	-	-
	LIPQNHAVIT	E3SH29; Q43608	Pru du 6	-	-	-
**AC10-GI**	HVSSDHNQLDQNPR	E3SH28; Q43607	Pru du 6	-	-	-
	FHVSSDHNQLDQNPR	E3SH28; Q43607	Pru du 6	-	-	-
	QQEQQQGQQGRPQ	E3SH28; Q43607	Pru du 6	-	-	-
	QQGEQGRPGQH	E3SH28; Q43607	Pru du 6	-	-	-
	HVSSDHNQLDQNP	E3SH28; Q43607	Pru du 6	-	-	-

TKSQTHVPIRPN	P82952	Pru du AP	-	-	-
VSSDHNQLDQNPR	E3SH28; Q43607	Pru du 6	-	-	-
QQGEQGRPGQHQ	E3SH28; Q43607	Pru du 6	-	-	-
QQQGQQGRPQ	E3SH28; Q43607	Pru du 6	-	-	-
QQGEQGRPGQHQQP	E3SH28; Q43607	Pru du 6	-	-	-
QEQQQGQQGRPQ	E3SH28; Q43607	Pru du 6	-	-	-
EQQQGQQGRPQ	E3SH28; Q43607	Pru du 6	-	-	-
QGQDDNRNEI	E3SH29; Q43608	Pru du 6	-	-	-
QQGEQGRPGQHQQPF	E3SH28; Q43607	Pru du 6	-	-	-
RGNLDFVQPPR	E3SH28; Q43607	Pru du 6	-	-	-
GNLDFVQPPR	E3SH28; Q43607	Pru du 6	-	-	-
GNLDFVQPPRG	E3SH28; Q43607	Pru du 6	-	-	-
EQEQQGGGGQD	E3SH29; Q43608	Pru du 6	-	-	-
QQGEQGRPGQ	E3SH28; Q43607	Pru du 6	-	-	-
QQGQQEQQQE	E3SH28; Q43607	Pru du 6	-	-	-
EHEERQQEQLQQE	E3SH28; Q43607	Pru du 6	-	-	-
IVPQNHGVIQQ	E3SH28; Q43607	Pru du 6	-	-	-
SQTHVPIRPN	P82952	Pru du AP	-	-	-
QQEQQGNGNNV	Q43607	Pru du 6	-	-	-
GEQGRPGQHQQPF	E3SH28; Q43607	Pru du 6	-	-	-
RQQQQQGEQG	E3SH28; Q43607	Pru du 6	-	-	-
AGNPQDEFNPQ	E3SH29; Q43608	Pru du 6	-	-	-
NENGDAILDQEVQQGQ	E3SH28; Q43607	Pru du 6	-	-	-
VTESWNPSDPQ	E3SH29; Q43608	Pru du 6	-	-	-
VRGNLDFVQPPR	E3SH28; Q43607	Pru du 6	-	-	-
ERQQQQQGEQG	E3SH28; Q43607	Pru du 6	-	-	-
RLKENIGNPE	E3SH28; Q43607	Pru du 6	-	-	-
DLNNDQNQLDQVPR	E3SH29; Q43608	Pru du 6	-	-	-
RTDENGFTNT	E3SH29; Q43608	Pru du 6	-	-	-
QQQEEGRQQE	E3SH28; Q43607	Pru du 6	-	-	-
REGDIIALPA	E3SH29; Q43608	Pru du 6	-	-	-
NENGDAILDQE	E3SH28; Q43607	Pru du 6	-	-	-

SFLRALPDEV	E3SH28; Q43607	Pru du 6	-	-	-
TIEPNGLHLPS	E3SH29	Pru du 6	-	-	-
TESWNPSDPQ	E3SH29; Q43608	Pru du 6	-	-	-
IQAEAGQIET	E3SH28; Q43607	Pru du 6	-	-	-
GEQGRPGQHQ	E3SH28; Q43607	Pru du 6	-	-	-
NPQDEFNPQQQGR	E3SH29; Q43608	Pru du 6	-	-	-
GEQGRPGQHQQP	E3SH28; Q43607	Pru du 6	-	-	-
IVPQNHGVIQ	E3SH28; Q43607	Pru du 6	-	-	-
RGNLDFVQPP	E3SH28; Q43607	Pru du 6	-	-	-
VRGNLDFVQPP	E3SH28; Q43607	Pru du 6	-	-	-
GQDDNRNEIV	E3SH29; Q43608	Pru du 6	-	-	-
FHVSSDHNQLDQNP	E3SH28; Q43607	Pru du 6	-	-	-
LANAYQISREQAR	E3SH28; Q43607	Pru du 6	-	-	-
RVQGQLDFVSP	E3SH29; Q43608	Pru du 6	-	-	-
NENGDAILDQ	E3SH28; Q43607	Pru du 6	-	-	-
QVVNENGDPI	E3SH29; Q43608	Pru du 6	-	-	-
QQQEEGRQQEQ	E3SH28; Q43607	Pru du 6	-	-	-
QGGGGQDNGVEET	E3SH29; Q43608	Pru du 6	-	-	-
IEPNGLHLPS	E3SH29	Pru du 6	-	-	-
QEQQGNGNNV	Q43607	Pru du 6	-	-	-
LAGNPQDEFN	E3SH29; Q43608	Pru du 6	-	-	-
RALPDEVLQNA	E3SH29	Pru du 6	-	-	-
LAGNPENEFN	E3SH28; Q43607	Pru du 6	-	-	-
RLSQNIGDPS	E3SH29; Q43608	Pru du 6	-	-	-
LDLNNDQNQLDQVPR	E3SH29; Q43608	Pru du 6	-	-	-
RGNLDFVQPPRG	E3SH28; Q43607	Pru du 6	-	-	-
TIALSSSQQR	E3SH28; Q43607	Pru du 6	-	-	-
TFEESQQSSQ	E3SH28; Q43607	Pru du 6	-	-	-
QREQEQQGGGGQD	E3SH29; Q43608	Pru du 6	-	-	-
EESQQSSQQG	E3SH28; Q43607	Pru du 6	-	-	-
NENGDAILDQEVQQG	E3SH28; Q43607	Pru du 6	-	-	-
YLAGNPQDEFN	E3SH29; Q43608	Pru du 6	-	-	-

	YLAGNPENEFN	E3SH28; Q43607	Pru du 6	-	-	-
	SRSAGGRGDQ	E3SH29; Q43608	Pru du 6	-	-	-
	NLQGQNDNRNQ	E3SH28; Q43607	Pru du 6	-	-	-
	LRALPDEVLA	E3SH28; Q43607	Pru du 6	-	-	-
	RNGLHLPSYS	E3SH28; Q43607	Pru du 6	-	-	-
	AILKDFDQPGT	Q8GSL5	Pru du 4	-	-	-
	TNANALVYAIRGN	E3SH29	Pru du 6	-	-	-
	AAVSCGQVVNNL	C0L0I5	Pru du 3	-	-	-
	AGLAAGLPGK	C0L0I5	Pru du 3	-	-	-
	GAGAGAAAPAAAEPK	Q8H2B9	Pru du 5	-	-	-
	YLAGNPQDEFNPQQQG	E3SH29; Q43608	Pru du 6	-	-	-
	IREGDIIALPA	E3SH29; Q43608	Pru du 6	-	-	-
	FIVPQNHGVIQ	E3SH28; Q43607	Pru du 6	-	-	-
	LAGNPENEFNQ	E3SH28; Q43607	Pru du 6	-	-	-
	VQGQLDFVSP	E3SH29; Q43608	Pru du 6	-	-	-
	LIPQNHAVIT	E3SH29; Q43608	Pru du 6	-	-	-
	QAGNQGFEYFA	E3SH28; Q43607	Pru du 6	-	-	-
	REGDVVAIPA	E3SH28; Q43607	Pru du 6	-	-	-
	PSGGGAVAVAA	Q8H2B9	Pru du 5	-	-	-
**H2O-AC10-GI**	HVSSDHNQLDQNPR	E3SH28; Q43607	Pru du 6	-	-	-
	HVSSDHNQLDQNP	E3SH28; Q43607	Pru du 6	-	-	-
	QGEDQQDRHQ	E3SH29; Q43608	Pru du 6	-	-	-
	VRGNLDFVQPPR	E3SH28; Q43607	Pru du 6	-	-	-
	QQGEQGRPGQHQ	E3SH28; Q43607	Pru du 6	-	-	-
	RNLQGQDDNRNEIV	E3SH29; Q43608	Pru du 6	-	-	-
	QQEQQQGQQGRPQ	E3SH28; Q43607	Pru du 6	-	-	-
	QQGEQGRPGQH	E3SH28; Q43607	Pru du 6	-	-	-
	TKSQTHVPIRPN	P82952	Pru du AP	-	-	-
	RNLQGQDDNRNEI	E3SH29; Q43608	Pru du 6	-	-	-
	DLNNDQNQLDQVPR	E3SH29; Q43608	Pru du 6	-	-	-
	FHVSSDHNQLDQNPR	E3SH28; Q43607	Pru du 6	-	-	-
	VSSDHNQLDQNPR	E3SH28; Q43607	Pru du 6	-	-	-

GNLDFVQPPR	E3SH28; Q43607	Pru du 6	-	-	-
SDHNQLDQNPR	E3SH28; Q43607	Pru du 6	-	-	-
QREQEQQGGGGQD	E3SH29; Q43608	Pru du 6	-	-	-
QQQGQQGRPQ	E3SH28; Q43607	Pru du 6	-	-	-
SSDHNQLDQNPR	E3SH28; Q43607	Pru du 6	-	-	-
GNLDFVQPPRG	E3SH28; Q43607	Pru du 6	-	-	-
QGQDDNRNEI	E3SH29; Q43608	Pru du 6	-	-	-
AGNPQDEFNPQ	E3SH29; Q43608	Pru du 6	-	-	-
DHNQLDQNPR	E3SH28; Q43607	Pru du 6	-	-	-
PILDDEVREGQ	E3SH29	Pru du 6	-	-	-
EQQQGQQGRPQ	E3SH28; Q43607	Pru du 6	-	-	-
QQGEQGRPGQHQQPF	E3SH28; Q43607	Pru du 6	-	-	-
RVQGQLDFVSP	E3SH29; Q43608	Pru du 6	-	-	-
SSDHNQLDQNP	E3SH28; Q43607	Pru du 6	-	-	-
RGNLDFVQPPR	E3SH28; Q43607	Pru du 6	-	-	-
QQGQQEQQQE	E3SH28; Q43607	Pru du 6	-	-	-
LDLNNDQNQLDQVPR	E3SH29; Q43608	Pru du 6	-	-	-
QQGEQGRPGQ	E3SH28; Q43607	Pru du 6	-	-	-
VRGNLDFVQPP	E3SH28; Q43607	Pru du 6	-	-	-
NENGDAILDQE	E3SH28; Q43607	Pru du 6	-	-	-
SQTHVPIRPN	P82952	Pru du AP	-	-	-
SDHNQLDQNP	E3SH28; Q43607	Pru du 6	-	-	-
QEQQQGQQGRPQ	E3SH28; Q43607	Pru du 6	-	-	-
QQGEQGRPGQHQQP	E3SH28; Q43607	Pru du 6	-	-	-
VTESWNPSDPQ	E3SH29; Q43608	Pru du 6	-	-	-
RALPDEVLQNA	E3SH29	Pru du 6	-	-	-
SQNIGDPSRAD	E3SH29; Q43608	Pru du 6	-	-	-
NGDPILDDEVR	E3SH29	Pru du 6	-	-	-
QQEQQGSGNNV	E3SH28	Pru du 6	-	-	-
HIREGDIIALPA	E3SH29; Q43608	Pru du 6	-	-	-
RQQQQQGEQG	E3SH28; Q43607	Pru du 6	-	-	-
EQEQQGGGGQD	E3SH29; Q43608	Pru du 6	-	-	-

GEQGRPGQHQQP	E3SH28; Q43607	Pru du 6	-	-	-
IVPQNHGVIQQ	E3SH28; Q43607	Pru du 6	-	-	-
PILDDEVREG	E3SH29	Pru du 6	-	-	-
GQDDNRNEIV	E3SH29; Q43608	Pru du 6	-	-	-
NENGDAILDQ	E3SH28; Q43607	Pru du 6	-	-	-
QGGGGQDNGVEET	E3SH29; Q43608	Pru du 6	-	-	-
TESWNPSDPQ	E3SH29; Q43608	Pru du 6	-	-	-
RGNLDFVQPP	E3SH28; Q43607	Pru du 6	-	-	-
REGDIIALPA	E3SH29; Q43608	Pru du 6	-	-	-
IVPQNHGVIQ	E3SH28; Q43607	Pru du 6	-	-	-
TIEPNGLHLPS	E3SH29	Pru du 6	-	-	-
IQAEAGQIET	E3SH28; Q43607	Pru du 6	-	-	-
RALPDEVLANA	E3SH28; Q43607	Pru du 6	-	-	-
QQQEEGRQQEQ	E3SH28; Q43607	Pru du 6	-	-	-
PILNDEVREGQ	Q43608	Pru du 6	-	-	-
AILDQEVQQGQ	E3SH28; Q43607	Pru du 6	-	-	-
VQGQLDFVSP	E3SH29; Q43608	Pru du 6	-	-	-
QQEEQQSQRE	E3SH29; Q43608	Pru du 6	-	-	-
NENGDAILDQEVQQGQ	E3SH28; Q43607	Pru du 6	-	-	-
QEQQGGGGQDN	E3SH29; Q43608	Pru du 6	-	-	-
GEQGRPGQHQQPF	E3SH28; Q43607	Pru du 6	-	-	-
EHEERQQEQLQQE	E3SH28; Q43607	Pru du 6	-	-	-
RLKENIGNPE	E3SH28; Q43607	Pru du 6	-	-	-
IREGDIIALPA	E3SH29; Q43608	Pru du 6	-	-	-
NENGDAILDQEVQQG	E3SH28; Q43607	Pru du 6	-	-	-
RLSQNIGDPS	E3SH29; Q43608	Pru du 6	-	-	-
STLNSHNLPI	E3SH28; Q43607	Pru du 6	-	-	-
SVVNRNHLPIL	E3SH29; Q43608	Pru du 6	-	-	-
IEPNGLHLPS	E3SH29	Pru du 6	-	-	-
SLLDLNNDQN	E3SH29; Q43608	Pru du 6	-	-	-
VVNENGDAILD	E3SH28; Q43607	Pru du 6	-	-	-
NLQGQNDNRNQ	E3SH28; Q43607	Pru du 6	-	-	-

NGDAILDQEV	E3SH28; Q43607	Pru du 6	-	-	-
EESQQSSQQG	E3SH28; Q43607	Pru du 6	-	-	-
ERQQQQQGEQG	E3SH28; Q43607	Pru du 6	-	-	-
NLQGQDDNRNE	E3SH29; Q43608	Pru du 6	-	-	-
SVVNRNHLPI	E3SH29; Q43608	Pru du 6	-	-	-
SLLDLNNDQNQ	E3SH29; Q43608	Pru du 6	-	-	-
LLDLNNDQNQLDQVPR	E3SH29; Q43608	Pru du 6	-	-	-
RTIEPNGLHLPS	E3SH29	Pru du 6	-	-	-
NPQGGRISVV	E3SH29; Q43608	Pru du 6	-	-	-
HLPSYSNAPQ	E3SH28; Q43607	Pru du 6	-	-	-
SRSAGGRGDQ	E3SH29; Q43608	Pru du 6	-	-	-
EPDNRIQAEAG	E3SH28; Q43607	Pru du 6	-	-	-
AQALNVNEET	E3SH28; Q43607	Pru du 6	-	-	-
VSLLDLNNDQN	E3SH29; Q43608	Pru du 6	-	-	-
LRALPDEVLA	E3SH28; Q43607	Pru du 6	-	-	-
ISVVNRNHLPI	E3SH29; Q43608	Pru du 6	-	-	-
TNANALVYAIRGN	E3SH29	Pru du 6	-	-	-
GVIQQAGNQGFE	E3SH28; Q43607	Pru du 6	-	-	-
YLAGNPQDEFNPQQQG	E3SH29; Q43608	Pru du 6	-	-	-
LIPQNHAVIT	E3SH29; Q43608	Pru du 6	-	-	-
YLAGNPQDEFN	E3SH29; Q43608	Pru du 6	-	-	-
YLAGNPENEFN	E3SH28; Q43607	Pru du 6	-	-	-
AGLAAGLPGK	C0L0I5	Pru du 3	-	-	-
AILDQEVQQG	E3SH28; Q43607	Pru du 6	-	-	-
VAVAAPGAGAGAAAP	Q8H2B9	Pru du 5	-	-	-
RQQQGGQLMANG	E3SH28	Pru du 6	-	-	-
VIQQAGNQGFE	E3SH28; Q43607	Pru du 6	-	-	-
TFEESQQSSQ	E3SH28; Q43607	Pru du 6	-	-	-
GNPQDEFNPQ	E3SH29; Q43608	Pru du 6	-	-	-
LAARQSQLSPQ	E3SH28; Q43607	Pru du 6	-	-	-
ALPDEVLQNA	E3SH29	Pru du 6	-	-	-
ILDDEVREGQ	E3SH29	Pru du 6	-	-	-

	QVVNENGDPI	E3SH29; Q43608	Pru du 6	-	-	-
	PSGGGAVAVAA	Q8H2B9	Pru du 5	-	-	-
	ISDQSKVGEQ	P82944	Pru du 2S Albumin	-	-	-
	EQEQQGGGGQ	E3SH29; Q43608	Pru du 6	-	-	-
	RALPDEVLAN	E3SH28; Q43607	Pru du 6	-	-	-
	ANAGLAAGLPGKCG	C0L0I5	Pru du 3	-	-	-
	QQQQQQQGQQGNGNN	E3SH29; Q43608	Pru du 6	-	-	-
